# Efficacy and Safety of Extracranial Vein Angioplasty in Multiple Sclerosis: A Randomized Clinical Trial

**DOI:** 10.1001/jamaneurol.2017.3825

**Published:** 2017-11-18

**Authors:** Paolo Zamboni, Luigi Tesio, Stefania Galimberti, Luca Massacesi, Fabrizio Salvi, Roberto D’Alessandro, Patrizia Cenni, Roberto Galeotti, Donato Papini, Roberto D’Amico, Silvana Simi, Maria Grazia Valsecchi, Graziella Filippini

**Affiliations:** 1 Translational Surgery and Vascular Diseases Centre, University of Ferrara Hospital, Ferrara, Italy; 2 Department of Biomedical Sciences for Health, Chair of Physical and Rehabilitation Medicine, University of Milan, Milan, Italy; 3 Italian Auxologico Institute, Milan, Italy; 4 Center of Biostatistics for Clinical Epidemiology, School of Medicine and Surgery, University of Milan-Bicocca, Milan, Italy; 5 Department of Neurosciences Drugs and Child Health, University of Florence, Florence, Italy; 6 Institute of the Neurological Science, Bellaria Hospital, Bologna, Italy; 7 Neuroradiology, Ravenna Hospital, Ravenna, Italy; 8 Interventional Radiology, University of Ferrara, Ferrara, Italy; 9 Regional Agency for Health and Social Care, Regione Emilia-Romagna, Italy; 10 Department of Diagnostic, Clinical and Public Health Medicine, University of Modena and Reggio Emilia, Modena, Italy; 11 MS Cochrane Group. Institute of Clinical Physiology, Pisa, Italy; 12 Scientific Director’s Office, Carlo Besta Foundation and Neurological Institute, Milan, Italy

## Abstract

**Importance:**

Chronic cerebrospinal venous insufficiency (CCSVI) is characterized by restricted venous outflow from the brain and spinal cord. Whether this condition is associated with multiple sclerosis (MS) and whether venous percutaneous transluminal angioplasty (PTA) is beneficial in persons with MS and CCSVI is controversial.

**Objective:**

To determine the efficacy and safety of venous PTA in patients with MS and CCSVI.

**Design, Setting, and Participants:**

We analyzed 177 patients with relapsing-remitting MS; 62 were ineligible, including 47 (26.6%) who did not have CCSVI on color Doppler ultrasonography screening. A total of 115 patients were recruited in the study timeframe. All patients underwent a randomized, double-blind, sham-controlled, parallel-group trial in 6 MS centers in Italy. The trial began in August 2012 and concluded in March 2016; data were analyzed from April 2016 to September 2016. The analysis was intention to treat.

**Interventions:**

Patients were randomly allocated (2:1) to either venous PTA or catheter venography without venous angioplasty (sham).

**Main Outcomes and Measures:**

Two primary end points were assessed at 12 months: (1) a composite functional measure (ie, walking control, balance, manual dexterity, postvoid residual urine volume, and visual acuity) and (2) a measure of new combined brain lesions on magnetic resonance imaging, including the proportion of lesion-free patients. Combined lesions included T1 gadolinium-enhancing lesions plus new or enlarged T2 lesions.

**Results:**

Of the included 115 patients with relapsing-remitting MS, 76 were allocated to the PTA group (45 female [59%]; mean [SD] age, 40.0 [10.3] years) and 39 to the sham group (29 female [74%]; mean [SD] age, 37.5 [10.6] years); 112 (97.4%) completed follow-up. No serious adverse events occurred. Flow restoration was achieved in 38 of 71 patients (54%) in the PTA group. The functional composite measure did not differ between the PTA and sham groups (41.7% vs 48.7%; odds ratio, 0.75; 95% CI, 0.34-1.68; *P* = .49). The mean (SD) number of combined lesions on magnetic resonance imaging at 6 to 12 months were 0.47 (1.19) in the PTA group vs 1.27 (2.65) in the sham group (mean ratio, 0.37; 95% CI, 0.15-0.91; *P* = .03: adjusted *P* = .09) and were 1.40 (4.21) in the PTA group vs 1.95 (3.73) in the sham group at 0 to 12 months (mean ratio, 0.72; 95% CI, 0.32-1.63; *P* = .45; adjusted *P* = .45). At follow-up after 6 to 12 months, 58 of 70 patients (83%) in the PTA group and 22 of 33 (67%) in the sham group were free of new lesions on magnetic resonance imaging (odds ratio, 2.64; 95% CI, 1.11-6.28; *P* = .03; adjusted *P* = .09). At 0 to 12 months, 46 of 73 patients (63.0%) in the PTA group and 18 of 37 (49%) in the sham group were free of new lesions on magnetic resonance imaging (odds ratio, 1.80; 95% CI, 0.81-4.01; *P* = .15; adjusted *P* = .30).

**Conclusion and Relevance:**

Venous PTA has proven to be a safe but largely ineffective technique; the treatment cannot be recommended in patients with MS.

**Trial Registration:**

clinicaltrials.gov Identifier: NCT01371760

## Key Points

**Question:**

What is the efficacy of venous percutaneous transluminal angioplasty (PTA) for chronic cerebrospinal venous insufficiency in patients with multiple sclerosis?

**Findings:**

In the Brave Dreams trial, which included 115 patients with relapsing-remitting multiple sclerosis, venous PTA did not increase the proportion of patients who improved functionally nor did it reduce the mean number of new combined brain lesions on magnetic resonance imaging at 12 months. However, there was a tendency for more patients to become free of new lesions after venous PTA mainly because of a reduction in new lesions appearing 6 to 12 months after randomization.

**Meaning:**

Venous PTA cannot be recommended for patients with relapsing-remitting multiple sclerosis.

## Teaser

This randomized clinical trial determines the efficacy and safety of venous percutaneous transluminal angioplasty in patients with multiple sclerosis with chronic cerebrospinal venous insufficiency.

## Introduction

When intraluminal defects, compression, or hypoplasia are identified in the internal jugular or azygos veins, the condition is known as chronic cerebrospinal venous insufficiency (CCSVI).^1^ An Italian open-label study^3^ published in 2009 including 65 patients with multiple sclerosis (MS) found an association between MS and CCSVI. The study also found that venous percutaneous transluminal angioplasty (PTA) was associated with an improved Multiple Sclerosis Functional Composite (MSFC) score at 1 year in patients with relapsing-remitting MS (RRMS) and a reduction in the proportion of those with gadolinium-enhancing lesions on brain magnetic resonance imaging (MRI). This preliminary study attracted considerable media attention in Italy and elsewhere; media-based groups formed to promote the treatment, and some called to make it freely available.^4^

Subsequent to the initial reports by Zamboni et al,^3^ prevalence studies of CCSVI in MS have reported conflicting results. A small 2012 case-control study^5^ further assessed the potential of venous PTA to benefit patients with MS. One year after venous PTA, the study found that functional score was improved compared with baseline, and the authors suggested that a double-blind randomized trial was justified.^5^

Both the National Institute for Health and Care Excellence and the Italian Ministry of Health have urged that randomized clinical trials be conducted to assess the efficacy of venous PTA for CCSVI in MS.^6^ In response to these calls and to public pressure, the Directorate-General for Health and Welfare of the Italian Region of Emilia Romagna funded the Brain Venous Drainage Exploited Against Multiple Sclerosis (Brave Dreams) trial to investigate the efficacy and safety of venous PTA in patients with MS and CCSVI. We report the results of that trial.

## Methods

### Study Design

The Brave Dreams trial was a multicenter, randomized, double-blind, sham-controlled, parallel-group trial to evaluate the efficacy and safety of venous PTA in patients with MS and CCSVI in extracranial or extravertebral veins. The study was conducted at 6 MS centers in Italy and their associated color Doppler ultrasonography (ECD) and angiography units, all of which were accredited by the Italian National Health Service. The Brave Dreams steering committee appointed an End Points Commission, which issued a detailed operations manual and trained trial physicians (who assessed primary functional outcomes, operated the ECD equipment, and performed catheter venography without venous angioplasty and venous PTA). After training, the commission issued an accreditation that was necessary for physicians to participate in the trial. Study monitoring was delegated to the company Medical Trials Analysis Italy in Ferrara, Italy. A trial data coordinating center was established to oversee data collection and quality and to perform the statistical analyses. The study adhered to the Helsinki Declaration and the International Council for Harmonisation of Technical Requirements for Pharmaceuticals for Human Use Good Clinical Practice guidelines and was approved by the ethical committee of the University of Ferrara Hospital and subsequently by the ethical committees of the other participating centers. The trial protocol has been published.^8^ The trial protocol can be found in Supplement 1. All participants provided written informed consent.

### Participants

Patients were recruited at the participating centers. Eligibility criteria included age 18 to 65 years; a diagnosis of RRMS, according to the 2005 McDonald criteria^9^; a diagnosis of secondary progressive MS, according to Lublin and Reingold^10^; care provided by the recruiting center for at least 2 years; at least 1 relapse in RRMS in the 2 years prior to enrollment; a baseline Expanded Disability Status Scale (EDSS) score of 2 to 5.5 ^11^; a disease duration of 15 years or less at baseline; stable neurological condition without relapse for at least 30 days before baseline; CCSVI, as determined by ECD examination carried out in accordance with a screening protocol^12^; and not receiving MS-specific treatment, immunomodulating, or immunosuppressive therapy without changes for at least 6 months up to baseline. Patients were ineligible if they had previous venous PTA or had received fingolimod therapy, cladribine therapy, laquinimod therapy, botulinum toxin therapy, infusion pump or neurostimulator implantation, or had participated in any clinical trial within 3 months of baseline. The complete list of exclusion criteria was published previously.^8^

### Randomization and Blinding

The data coordinating center set up an internet-based computerized central randomization protocol stratified by participating center with variable length blocks, which assigned patients to the PTA or sham group in a 2:1 ratio. Treatment assignment was made known to the treating surgeon (via the electronic case report form) only on the day of the operation. Patients, all other study investigators, and operating room and hospital personnel were blinded to assignment.

To maintain patient blinding, surgeons were trained to deliver a catheter venography intervention that simulated venous PTA.^8^ This involved sudden acceleration of the catheter as it passed through the internal jugular vein together with a comment from the radiologist suggesting that venous PTA had been performed.

### Procedures

Participants underwent catheter venography without venous angioplasty of the azygos and internal jugular veins, with percutaneous access via the left femoral vein. The presence and location of CCSVI was assessed as reported elsewhere.^2^ If venography was positive for CCSVI, participants randomized to the PTA group received venous PTA during the venography session. If CCSVI was absent, those assigned to the PTA group received catheter venography without venous angioplasty. Those allocated to the sham group received catheter venography without venous angioplasty. These procedures were performed via day surgery. Overnight hospital stay was never required in this trial. All patients received prophylactic low-molecular-weight heparin during the 3 subsequent weeks.

### Outcomes

There were 2 primary end points at 12 months: a functional end point and an MRI end point.

#### Functional End Point

In view of the psychometric limitations of the EDSS and the MSFC scores,^13^ the steering committee decided to use a new primary composite end point based on a range of the functional impairments commonly experienced by patients with MS. This composite included walking control, balance, manual dexterity, postvoid residual urine volume, and visual acuity (eMethods in Supplement 2). Statistically significant changes were adopted for defining changes in walking control, balance, and manual dexterity; a minimal real difference was used for postvoid residual urine volume and visual acuity.^15^ Based on the changes found, each index was considered improved, stable, or worsened. Patients were classified as (1) improved, which indicated improvement in 1 or more functions and stability in the remaining functions, (2) worsened, which indicated worsening of 1 or more functions and stability in nonworsening functions, (3) mixed, which indicated presence of improved and worsened functions, or (4) stable, which indicated no change in any function. Evaluations were performed by operators (2 per participating center) at baseline, within 15 days of venography, and 3, 6, and 12 months later. Only results at 12 months were used in analyses. To ensure the reliability of the functional measurements, the End Points Commission made 1 or 2 visits to each site to monitor the functional assessments. Assessors were required to make video recordings of the walking control and manual dexterity tests in 10% or more of patients and to send them to the commission for inspection.

If a patient had a transient impairment that blocked performance of 1 or more functional tests, these were not performed until the next follow-up. In the event of a clinical relapse, all tests were deferred. As per study protocol, the final round of tests could take place up to 15 months after venography.

#### Magnetic Resonance Imaging End Point

Scans were acquired at baseline and at 6 and 12 months after venography. The primary MRI end point was the number of new combined cerebral lesions at 12 months compared with baseline. New combined lesions included (1) new lesions on T2-weighted images, (2) preexisting lesions enlarged by greater than 30% on T2-weighted images, and (3) gadolinium-enhancing lesions in T1-weighted images of preexisting lesions. An additional MRI end point was the proportion of patients free of new lesions. Before starting the study, each center acquired a set of MRI scans of a single individual on 2 separate occasions. The scans were assessed by the Department of Neurosciences at the University of Florence for image quality, repositioning accuracy, and signal-to-noise ratio to check that quality was sufficient. At each center, MRIs were always performed with the same device (at least 1.5 T) and with the same protocol. The scans were assessed by an experienced specialist blinded to treatment assignment.

### Secondary Outcomes

Secondary outcomes at 12 months were the proportion of patients with CCSVI diagnosed by ECD but not confirmed by venography, annualized relapse rate, change in EDSS score, proportion of patients with relapses, and proportion of patients who had venous PTA with restored flow on ECD at 12 months.

### Safety

The types and grade of adverse events are reported according to the Good Clinical Practice guidelines. Stopping rules included serious adverse events and/or a false-positive rate for CCSVI on ECD exceeding 10%.^8^

### Statistical Analysis

To achieve a 90% power (SD = 7.6) to detect a hypothesized 2.1 fewer active MRI lesions in the PTA group (3.9 vs 6.0), 423 patients with RRMS were needed. This number of patients was also sufficient (92% power) to detect a hypothesized 15% more patients in the PTA group than the sham group improving on the functional end point (α = .05; 2-sided test on proportions). Patients in the sham group were themselves expected to improve by 15% over the year of follow-up. Because both primary end points were used to characterize treatment benefit, each null hypothesis had to be rejected at the same significance level (α = .05), with multiplicity adjustment only for the MRI end point, since this had 2 parameters (mean lesion number and proportion of lesion-free patients).^16^

Because of low enrollment (6 patients per month by November 2013), the protocol was amended in February 2014; 300 patients with RRMS (200 in PTA group and 100 in sham group) were required to reveal the same hypothesized differences in MRI and functional end points, with powers of 80% and 84%, respectively. The study closed in December 2014 because of slow recruitment, with 115 patients with RRMS recruited (38.3% of amended target).

The initial power calculation for patients with secondary progressive MS indicated that 222 patients were needed.^8^ Recruitment was stopped in February 2014, with only 15 patients recruited. Stopping decisions were taken without knowledge of outcomes or treatment allocations.

For descriptive purposes, we calculated percentage, mean (SD), and median (interquartile range), as appropriate. The main analysis was intention to treat. The effect of PTA vs sham on the composite functional end point was assessed by comparing the proportions of improved patients at 1 year in the 2 groups, and the significance of differences in proportion was assessed by χ^2^ test. The effect of PTA vs sham on the MRI end point was assessed by a negative binomial model that compared the mean number of MRI lesions in the 2 groups at 1 year. The proportion of patients who were free of new brain lesions on MRI were compared by χ^2^ test. Variables that were unbalanced at baseline were adjusted for using a logistic model on the functional end point and a negative binomial model on mean number of MRI lesions.

Relapse rates (secondary end point) in the 2 groups were compared assuming a Poisson distribution of events. Differences in EDSS scores at 1 year were compared by analysis of covariance testing, with adjustment for baseline scores. When analyzing components of the functional and MRI end points (including findings at 0 to 6 and 6 to 12 months), we adjusted for multiplicity using the Hommel method,^17^ since it is reasonable to assume that variables involved could be directly related (reported as adjusted *P* value). All tests were 2-tailed, with the significance level set at *P* < .05. Analyses were performed with SAS version 9.3 (SAS Institute) and R version 3.2 (R Foundation for Statistical Computing).

## Results

The Figure shows the study flowchart for patients with RRMS. A total of 177 were assessed for eligibility, and 62 were ineligible, including 47 (26.6%) who did not have CCSVI on ECD screening. One hundred fifteen patients were eligible and randomly assigned to the PTA group (n = 76) or the sham group (n = 39), which included catheter venography without venous angioplasty, between August 7, 2012, and December 15, 2014. The 2 groups were similar for baseline characteristics except that patients in the sham group had more women and longer disease duration (Table 1). No serious adverse events attributable to catheter venography or venous PTA occurred, but 2 adverse events (1.7%) did occur: 1 vagal reaction and 1 episode of transient neck pain. A total of 112 of 115 patients (97.4%) completed the 12-month follow-up, with similar proportions completing in the 2 groups. The eFigure in Supplement 2 shows the study flowchart for patients with secondary progressive MS.

**Figure.**
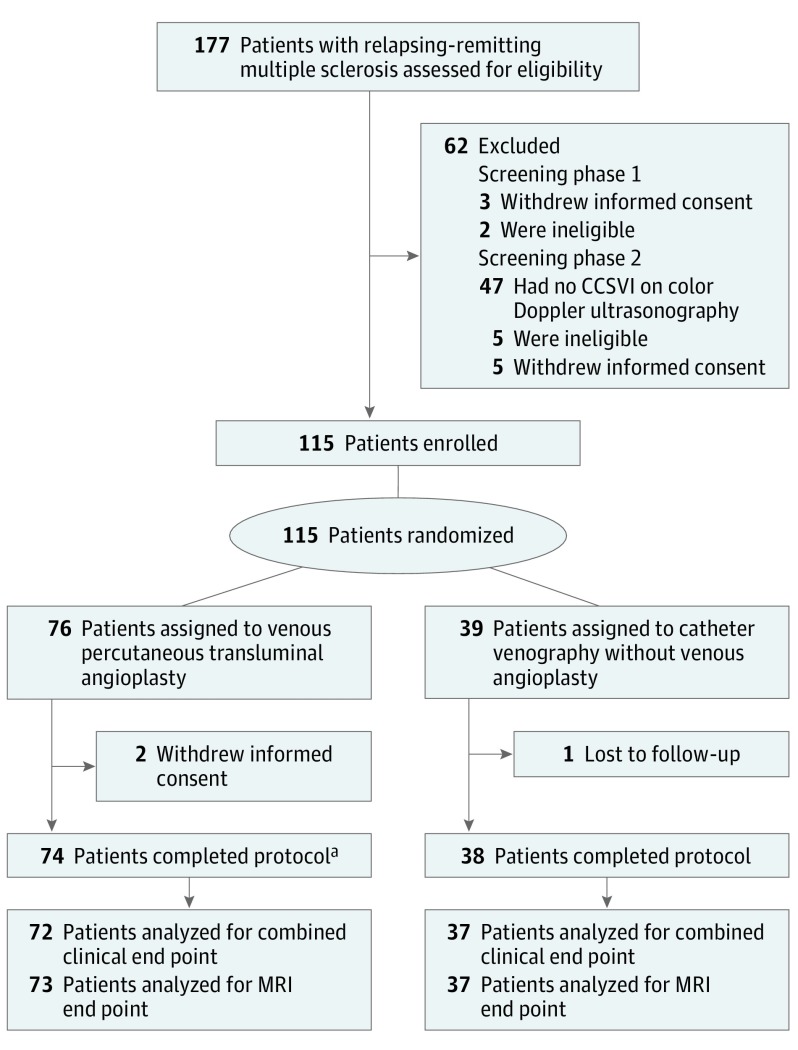
Study Flowchart for Patients With Relapsing-Remitting Multiple Sclerosis ^a^Includes 2 patients who received the sham treatment; one patient was included by mistake and the other was included because chronic cerebrospinal venous insufficiency was not confirmed on venography. CCSVI indicates chronic cerebrospinal venous insufficiency; MRI, magnetic resonance imaging.

**Table 1.** Baseline Characteristics of Recruited Patients and Their Disease According to Treatment Group

Characteristic	No. (%)	
	PTA (n = 76)	Sham (n = 39)
Female	45 (59)	29 (74)
Age, mean (SD), y	40.0 (10.3)	37.5 (10.6)
EDSS score		
2 or 2.5	50 (66)	24 (62)
3 or 3.5	18 (24)	11 (28)
4 or 4.5	8 (11)	2 (5)
5 or 5.5	0	2 (5)
Median (interquartile range)	2.5 (2.0-3.0)	2.5 (2.0-3.5)
Mean (SD)	2.6 (0.7)	2.7 (0.9)
Years since MS diagnosis, median (interquartile range)	4.3 (2.8-8.4)	6.1 (3.7-9.0)
Relapses in previous 2 years, No.		
0	0	0
1	44 (58)	26 (67)
2	24 (32)	4 (10)
≥3	8 (11)	9 (23)
Median (interquartile range)	1.0 (1.0-2.0)	1.0 (1.0-2.0)
Mean (SD)	1.7 (1.2)	1.8 (1.4)
Intraluminal obstacle at ECD in at least 1 IJV		
Yes	74 (97)	37 (95)
No	2 (3)	2 (5)
Bidirectional and/or absent flow at ECD in at least 1 IJV, in 2 positions		
Yes	70 (92)	38 (97)
No	6 (8)	1 (3)
T1 gadolinium-enhancing lesions, No.		
Median (range)	0 (0-8)	0 (0-3)
Mean (SD)	0.49 (1.2)	0.23 (0.58)
Immunomodulatory therapy		
Yes	31 (41)	18 (46)
No	45 (59)	21 (54)

Abbreviations: ECD, color Doppler ultrasonography; EDSS, Expanded Disability Status Scale; IJV, internal jugular vein; MS, multiple sclerosis; PTA, percutaneous transluminal angioplasty.

### Primary End Points

Functional end point results were available for 109 patients with RRMS (Table 2). A total of 30 of 73 patients (41%) in the PTA group and 18 of 37 (49%) in the sham group improved on the functional end point—a difference of −7% (95% CI, −26.7 to 10.1) in favor of the sham group. The logistic model adjusted for sex and disease duration provided an odds ratio (OR) for improvement in the PTA group (compared with the sham group) of 0.70 (95% CI, 0.31-1.59; *P* = .40). The OR for the unadjusted model was 0.75 (95% CI, 0.34-1.68; *P* = .49). Worsening occurred in 9 patients (12%) in the PTA group vs 7 (19%) in the sham group; functional stability was maintained in 17 (23%) in the PTA group and 8 (22%) in the sham group. A mixed outcome (improvement in one or more functions and worsening in one or more) occurred in 16 patients (22%) in the PTA group and 4 (11%) in the sham group. Results for individual components of the functional end point are shown in Table 3. More patients in the PTA group than in the sham group improved in visual acuity and manual dexterity, while more patients in the PTA group worsened for postvoid residual urine volume and balance. Walking control remained stable in most patients of both groups.

**Table 2.** Results for Components of Composite Functional End Point and Magnetic Resonance Imaging (MRI) End Point

Finding	No. (%)		Unadjusted Estimated Effect of Venous PTA, OR (95% CI)	*P* Value	Adjusted *P* Value^a^
	PTA (n = 73)	Sham (n = 37)			
Composite functional end point^b^					
Improved	30 (42)	18 (49)	0.75 (0.34-1.68)^c^	.49	NA
Stable	17 (24)	8 (22)	NA		
Worsened	9 (13)	7 (19)	NA		
Mixed	16 (22)	4 (11)	NA		
MRI end point (new combined lesions)^d^					
No. of lesions, mean (SD)	1.40 (4.21)	1.95 (3.73)	0.72 (0.32-1.63)^e^	.45	.45
Median (range)	0 (0-31)	1 (0-8)	NA	NA	NA
No. patients free of new lesions	46 (63)	18 (49)	1.80 (0.81-4.01)^f^	.15	.30

Abbreviations: NA, not applicable; OR, odds ratio; PTA, percutaneous transluminal angioplasty.

^a^ *P* values adjusted for multiplicity with Hommel method.^17^

^b^ Seventy-two patients in the PTA group had composite functional end point data.

^c^ OR for PTA group improvement with 95% CI and *P* value from logistic model.

^d^ New combined lesions include new lesions on T2-weighted images, preexisting lesions enlarged by >30% on T2-weighted images, and gadolinium-enhancing lesions in T1-weighted images of preexisting lesions (no enlarged T2 lesions were observed).

^e^ Mean lesion ratio with 95% CI and *P* value from negative-binomial model.

^f^ OR of being lesion free with 95% CI and *P* value from χ^2^ test.

**Table 3.** Detailed Results for Components of Primary Functional End Point^a^

Functional Assessment	Total No.	At 12 mo, No. (%)			Baseline Score		Score at 12 mo	
		Improved	Stable	Worsened	Median (Range)	Mean (SD)	Median (Range)	Mean (SD)
**Visual Acuity (No. of Lines Read)**								
100% Contrast								
PTA	72	5 (7)	64 (89)	3 (8)	12.0 (8.0-14.0)	11.9 (1.0)	12.0 (9.0-14.0)	11.9 (1.0)
Sham	37	1 (3)	33 (89)	3 (4)	12.0 (6.0-14.0)	11.9 (1.6)	12.0 (6.0-14.0)	11.6 (1.7)
2.5% Contrast								
PTA	72	6 (8)	61 (85)	5 (7)	8.0 (3.0-10.0)	7.5 (1.3)	8.0 (5.0-10.0)	7.64 (1.2)
Sham	37	1 (3)	33 (89)	3 (8)	8.0 (3.0-10.0)	7.3 (1.7)	8.0 (1.0-10.0)	7.22 (1.9)
1.25% Contrast								
PTA	72	11 (15)	59 (82)	2 (3)	6 (1.0-8.0)	5.7 (1.4)	6.0 (3.0-9.0)	6.00 (1.5)
Sham	37	4 (11)	30 (81)	3 (8)	6 (0.0-8.0)	5.3 (2.0)	5.0 (0.0-10.0)	5.41 (2.3)
Any contrast								
PTA	72	15 (21)	49 (68)	8 (11)	NA	NA	NA	NA
Sham	37	5 (14)	27 (73)	5 (14)	NA	NA	NA	NA
**Manual Dexterity (No. of Cubes Displaced)**								
Dominant hand								
PTA	72	13 (18)	55 (76)	4 (6)	67.3 (38.0-81.0)	64.5 (10.1)	68.3 (40.5-89.5)	67.7 (10.1)
Sham	37	5 (14)	31 (84)	1 (3)	66.0 (36.0-85.5)	64.4 (11.4)	67.0 (42.0-92.5)	67.0 (13.1)
Nondominant hand								
PTA	72	14 (19)	55 (76)	3 (4)	63.5 (35.0-77.0)	61.6 (9.3)	64.8 (31.5-83.0)	64.4 (11.3)
Sham	37	6 (16)	30 (81)	1 (3)	61.0 (42.5-86.0)	62.4 (10.9)	63.0 (38.0-89.0)	64.8 (12.8)
Any hand								
PTA	72	18 (25)	50 (69)	4 (6)	NA	NA	NA	NA
Sham	37	7 (19)	29 (78)	1 (3)	NA	NA	NA	NA
**Postvoid Residual Urine Volume (mL)**								
PTA	69	20 (30)	36 (54)	11 (16)	43.0 (0.0-256.0)	64.7 (61.4)	33.0 (0.0-327.0)	50.3 (58.9)
Sham	36	11 (31)	22 (61)	3 (8)	54.0 (0.0-263.0)	73.2 (69.1)	35.5 (0.0-277.0)	57.3 (64.4)
**Balance Test (% Adherence to Path)**								
PTA	72	16 (22)	51 (71)	5 (7)	84.0 (46.0-109.0)	82.5 (12.0)	88.5 (54.0-102.0)	85.0 (10.2)
Sham	37	8 (22)	28 (76)	1 (3)	81.0 (60.0-99.0)	78.6 (11.7)	84.0 (60.0-102.0)	82.2 (11.8)
**Walking Control (Walk Ratio** ^b^ **)**								
PTA	72	0	70 (97)	2 (3)	5.5 (4.2-7.6)	5.6 (0.8)	5.5 (4.2-7.5)	5.6 (0.8)
Sham	37	0	36 (97)	1 (3)	5.4 (3.7-7.3)	5.3 (0.8)	5.3 (3.6-7.1)	5.3 (0.8)

Abbreviations: NA, not applicable; PTA, percutaneous transluminal angioplasty.

^a^ All *P* values were >.99 after adjustment for multiplicity.

^b^ Ratio of step length (millimeters) to step frequency (per minute).

The number of new lesions on MRI at 12 months compared with baseline was unrelated to venous PTA; there were a mean 1.40 new lesions in the PTA group vs 1.95 in the sham group (mean ratio, 0.72; 95% CI, 0.32-1.63; *P* = .45; adjusted *P* = .45) (Table 2). In the regression analysis adjusted for sex and disease duration, the mean PTA-to-sham ratio was 0.50 (95% CI, 0.20-1.27; *P* = .30). The proportion of patients free of new lesions did not differ significantly between the PTA and sham groups (63.0% vs 48.6%; OR, 1.80; 95% CI, 0.81-4.00; *P* = .15; adjusted *P* = .30) (Table 2). At 12 months, 50 patients (68%) in the PTA group were free of new or enlarged T2 lesions compared with 21 (57%) in the sham group (OR, 1.66; 95% CI, 0.73-3.75; *P* = .22; adjusted *P* = .62), and 53 (73%) in the PTA group were free of gadolinium-enhancing lesions at 12 months compared with 18 (49%) in the sham group (OR, 2.76; 95% CI, 1.14-6.68; *P* = .02; adjusted *P* = .08) (Table 4).

**Table 4.** Magnetic Resonance Imaging Findings at 6 to 12 Months and 0 to 12 Months

Measure	Months 6-12				Months 0-12			
	PTA (n = 70)	Sham (n = 33)	*P* Value	Adjusted *P* Value^a^	PTA (n = 73)	Sham (n = 37)	*P* Value	Adjusted *P* Value^a^
New combined brain lesions^b^								
No. of lesions, mean (SD)	0.47 (1.19)	1.27 (2.65)	NA	NA	1.40 (4.21)	1.95 (3.73)	NA	NA
Mean lesion ratio (95% CI)^c^	0.37 (0.15-0.91)		.03	.09	0.72 (0.32-1.63)		.45	.45
Median (range)	0 (0-7)	0 (0-13)	NA	NA	0 (0-31)	1 (0-8)	NA	NA
Patients free of lesions, No. (%)	58 (79.5)	22 (59.5)	NA	NA	46 (63.0)	18 (48.7)	NA	NA
Lesion free, OR (95% CI)^d^	2.64 (1.11-6.28)		.03	.09	1.80 (0.81-4.01)		.15	.30
New or enlarged T2 lesions								
No. of lesions, mean (SD)	0.36 (0.98)	1.14 (2.63)	NA	NA	1.10 (3.03)	1.59(3.42)	NA	NA
Mean lesion ratio (95% CI)^c^	0.31 (0.11-0.86)		.02	.06	0.69 (0.28-1.67)		.41	.82
Median (range)	0 (0-5)	0 (0-13)	NA	NA	0	0 (0-18)	NA	NA
Patients free of lesions, No. (%)	61 (83.6)	24 (64.9)	NA	NA	50 (68.5)	21 (56.8)	NA	NA
Lesion free, OR (95% CI)^d^	2.75 (1.10-6.89)		.03	.09	1.66 (0.73-3.75)		.22	.62
Gadolinium-enhancing T1 lesions								
No. of lesions, mean (SD)	0.36 (1.08)	0.59 (1.55)	NA	NA	1.07 (4.11)	1.06 (2.47)	NA	NA
Mean lesion ratio (95% CI)^c^	0.60 (0.20-1.75)		.35	.35	1.01 (0.35-2.93)		.98	.98
Median (range)	0 (0-7)	0 (0-9)	NA	NA	0	0 (0-14)	NA	NA
Patients free of lesions, No. (%)	62 (84.9)	26 (70.3)	NA	NA	53 (76.8)	18 (54.6)	NA	NA
Lesion free, OR (95% CI)^d^	2.38 (0.92-6.19)		.07	.14	2.76 (1.14-6.68)		.02	.08

Abbreviations: NA, not applicable; OR, odds ratio; PTA, percutaneous transluminal angioplasty.

^a^ *P* values adjusted for multiplicity using Hommel method.^17^

^b^ New combined lesions included new lesions on T2-weighted images, preexisting lesions enlarged by >30% on T2-weighted images, and gadolinium-enhancing lesions in T1-weighted images of preexisting lesions (no enlarged T2 lesions were observed).

^c^ Estimated effect of PTA on number of lesions; negative-binomial model was used to derive *P* values.

^d^ Estimated effect of PTA on proportion lesion-free patients; χ^2^ test was used to derive *P* values.

A post hoc analysis investigating new brain lesions at 0 to 6 months (eTable 1 in Supplement 2) and at 6 to 12 months found that at 6 to 12 months, 58 of 70 patients (83%) in the PTA group and 22 of 33 (67%) in the sham group (OR, 2.64; 95% CI, 1.11-6.28; *P* = .03; adjusted *P* = .09) were free of combined brain lesions, while there were a mean (SD) 0.36 (0.98) new T2 lesions in the PTA group and 1.14 (2.63) in the sham group (*P* = .02; adjusted *P* = .06). Finally, 61 patients (87%) in the PTA group and 24 (73%) in the sham group (OR, 2.75; 95% CI, 1.10-6.89; *P* = .03; adjusted *P* = .09) were free of new T2 lesions (Table 4).

### Secondary End Points

Eight of 115 patients with RRMS (7.0%) diagnosed as having CCSVI by ECD had no abnormalities on venography, so the positive predictive value of ECD was 93.0% in patients with RRMS. The annualized relapse rate was 0.32 (95% CI, 0.2-0.4) in the PTA group and 0.39 (95% CI, 0.2-0.5) in the sham group, giving a relative rate of 0.82 (95% CI, 0.40-1.71; *P* = .60). The median (interquartile range) baseline EDSS score was 2.5 (2.0-3.0) in the PTA group and 2.5 (2.0-3.5) in the sham group (Table 1). At 12 months, the median (interquartile range) EDSS score was 2.0 (1.5-3.0) in the PTA group and 2.0 (1.5-2.5) in the sham group (*P* = .49). Seventeen of 73 patients (23%) in the PTA group had least 1 relapse over the 12 months compared with 12 of 39 (31%) in the sham group. Blinded flow assessment at 12 months revealed restored flow in 38 of 71 patients (54%) in the PTA group and 14 of 37 (38%) in the sham group.

### Secondary Progressive MS

Primary end points for the 15 patients with secondary progressive MS (10 in the PTA group and 5 in the sham group) are shown, in descriptive form only, in eTable 2 in Supplement 2.

## Discussion

Venous PTA did not increase the proportion of patients with RRMS who improved on the functional composite measure compared with the sham procedure over the 12-month follow-up, nor did it significantly reduce the appearance of new combined brain lesions on MRI at 0 to 12 months. Expanded Disability Status Scale disability measures were stable and similar between the groups at 12 months, matching the composite functional outcome. The annualized relapse rate was also similar between the groups. Safety data indicated no serious adverse events attributable to venous PTA or the sham procedure.

The functional outcome explored by the trial was reduction in disability as assessed by 5 functions (ie, walking control, balance, manual dexterity, postvoid residual urine volume, and visual acuity; eMethods in Supplement 2), which are the most frequent causes of disability in MS. The hypothesis that venous PTA can significantly reduce disability is rejected by findings from this study, which not only found no difference between the groups on the functional composite measure but also no difference for any of its 5 components (Table 3).

The primary MRI outcome measure was a difference in the number of new combined brain lesions. Venous PTA had no effect on this measure (Table 2). However, at 12 months, more than 20% of patients in the PTA group were free of gadolinium-enhancing lesions compared with the sham group (OR, 2.76; 95% CI, 1.14-6.68) (Table 4). To explore this effect, which appears inconsistent with the other MRI data at 0 to 12 months, we performed an exploratory post hoc comparison of MRI findings at 0 to 6 months with those at 6 to 12 months (Table 4; eTable 1 in Supplement 2). We found a reduction in the mean number of new brain lesions (corresponding to more lesion-free patients) in the PTA group compared with the sham group at 6 to 12 months. The delayed and positive effect on the magnetic resonance biomarker suggests that PTA could affect the dynamic of the blood-brain barrier. 

Gadolinium enhancement is a marker of damage to the blood-brain barrier, whose time course depends on lymphatic drainage^18^ and hence on venous drainage from the skull.^19^ Previous studies have reported that venous pressure is lowered^3^ and cerebrospinal fluid dynamics is improved^20^ after venous PTA, thereby favoring the drainage of cerebrospinal fluid into the dural veins, which depends on a pressure gradient between the subarachnoid spaces and dural veins.^21^ Another study^23^ reported that white matter lesion load was inversely correlated with reduced cerebrospinal fluid dynamics, as measured by MRI. In addition, flow improvement through the internal jugular veins owing to venous PTA has been reported to improve brain perfusion in patients with RRMS.^21^ It has also been reported that the development of a new MS plaque was preceded by sustained MRI-detected hypoperfusion before the plaque was identified on MRI.^24^

To our knowledge, few published data are available to compare with Brave Dreams findings. The positive effect of venous PTA on disability found in the first open-label pilot study^3^ was not confirmed in the present study. The first study^3^ was not blinded and used the MSFC and the EDSS scores as primary end points. There is some degree of subjectivity in assessing MSFC and EDSS scores, and with nonblinded patients and outcome assessors, detection bias may have been introduced.

In a sham-controlled randomized trial,^26^ 19 patients with MS (9 assigned to PTA and 10 to sham) were assessed 1, 3, and 6 months after intervention. It was found that clinical and MRI end points were closely similar in the 2 groups. However, the study was flawed by the small number of recruited patients and short follow-up.^26^ In an observational study,^27^ 29 patients with RRMS were observed for 2 years after venous PTA.A small improvement in mean EDSS score occurred over that time, and the mean annual relapse rate also lowered. Magnetic resonance imaging data were not reported.

### Limitations

Our study has several limitations. Despite being, to our knowledge, the largest randomized study on venous PTA in RRMS, the power of the study was limited. There are at least 3 main reasons for this. First, many MS centers are reluctant to propose patients for a trial exploring a controversial hypothesis. Thus, of the 15 centers that initially joined the trial, only 6 actively recruited. Second, many patients are reluctant to participate in a randomized sham-controlled study in spite of the fact that randomization was 2:1 in favor of angioplasty; this reluctance may have been exacerbated by the media, which overemphasized the effectiveness of venous PTA. Third, the sudden widespread availability of venous PTA (and stenting) in private centers decreased the number of patients fulfilling the inclusion criterion of no previous angioplasty. A post hoc power calculation, based on control findings, indicated a power of 30% to detect a difference in the functional end point and 17% power to detect the target difference in the MRI end point. In terms of effect size and clinical relevance, the 95% CI for the estimated 7% advantage of sham varied from 26.7% in favor of sham to 10.1% in favor of PTA, thereby excluding the 15% target threshold for a benefit of venous PTA on the functional end point. However, the findings on new MRI lesions do not completely exclude an effect of venous PTA, since the 95% CI for the mean lesion ratio of 0.72 in favor of PTA includes the 0.65 target for PTA improvement (95% CI, 0.32-1.63).

The use of a new composite functional outcome measure was a major challenge. Although the outcomes and change thresholds (reflecting sensitivity to change) of the individual components have been validated (eMethods in Supplement 2), the composite is presented for the first time in this article.

Another limitation of the trial is that the severity of the enrolled patients in terms of EDSS score and lesion load was lower than anticipated, and patients with active disease may have been underrepresented in the study (Table 1). Finally, the fact that venous PTA was largely ineffective in restoring blood flow in nearly half the patients in the PTA group suggests that it was inadequate for exploring our initial hypothesis.

## Conclusions

A number of neurologists and scientists expressed the opinion that the decision to conduct a trial on CCSVI in the absence of valid scientific evidence was unethical and a waste of resources.^28^ However, we believe that the best way to provide useful information to patients (and regulatory authorities) on the benefit and safety of venous PTA was to conduct a randomized trial—as also recommended by NICE^7^—that assessed outcomes directly relevant to patients.^29^ Venous PTA has proven to be a safe but ineffective technique in treating CCSVI in about half of patients. The procedure cannot be recommended for treatment of patients with MS; no further double-blinded clinical studies are needed. The delayed effect of venous PTA 6 months after the procedure on the magnetic resonance biomarker suggests a possibility that PTA may produce benefit for a subgroup of patients with MS. This should be further analyzed and investigated.

## Notes

Supplement 1. Trial Protocol

Supplement 2. **eMethods.** Information on the novel functional composite. **eFigure.** Study diagram for patients with secondary progressive multiple sclerosis. **eTable 1.** Magnetic resonance imaging findings at 0 to 6 months. **eTable 2.** Clinical and magnetic resonance imaging end points (changes at 12 months compared with baseline) according to treatment group in the 15 patients with secondary progressive multiple sclerosis (intention-to-treat population).
