# Supplementary material for: Efficacy and Safety of Extracranial Vein Angioplasty in Multiple Sclerosis: A Randomized Clinical Trial
Source: JAMA Neurol. 2017 Nov 18;75(1):35–43. doi: 10.1001/jamaneurol.2017.3825 (PMC5833494; doi:10.1001/jamaneurol.2017.3825)
Supplement: Supplement 1. — Trial Protocol [file jamaneurol-75-35-s001.pdf]

Studio CCSVI-SM: efficacia e sicurezza dell'angioplastica venosa nella SM

STUDY PROTOCOL

Title:

Randomized multi-centered study for evaluating the efficacy and safety of angioplastic surgery of the extracranial veins in the treatment of multiple sclerosis

BRAVE DREAMS (BRAin VEnous DRainage Exploited Against Multiple Sclerosis)

Short title:

CCSVI-SM Study: efficacy and safety of venous angioplasty in MS

Version April 26, 2011

Coordination and Promotion Center:

- Azienda Ospedaliero Universitaria di Ferrara

PI: Paolo Zamboni: Direttore Centro Malattie Vascolari, Università degli Studi di Ferrara, Unità Operativa Clinica Chirurgica, Azienda Ospedaliero-Universitaria Arcispedale "S.Anna", Ferrara

Executive Steering Committee:

- **Graziella Filippini:** Neurologo – Metodologo, IRCCS "C. Besta, Milano/ Gruppo Cochrane Internazionale Sclerosi Multipla
- **Alessandro Liberati:** Epidemiologo-Metodologo, Università di Modena e Reggio Emilia/Assr – Regione Emilia Romagna, Bologna
- **Luigi Tesio:** Fisiatra, Università di Milano, IRRCS Auxologico
- **Paolo Zamboni:** Chirurgo Vascolare, Azienda Ospedaliero Universitaria di Ferrara
- **Patrizia Cenni:** Neuroradiologo, Azienda USL di Ravenna, Ospedale di Ravenna

Steering Committee Chairperson:

- **Graziella Filippini:** Neurologo **–** Metodologo, IRCCS "C. Besta, Milano/ Gruppo Cochrane Internazionale Sclerosi Multipla

Protocol drafting and updating:

- **Donato Papini:** Epidemiologo, Assr- Regione Emilia Romagna, Bologna
- **Alessandro Liberati:** Epidemiologo-Metodologo, Università di Modena e Reggio Emilia/Assr – Regione Emilia Romagna, Bologna

Clinical Endpoint Commission (CEC):

- **Antonio Bertolotto:** Neurologo, Azienda Ospedaliero-Universitaria San L.Gonzaga, Orbassano (TO)
- **Paolo Boldrini:** Fisiatra, ULSS 9 Traviso – Ospedale Riabilitativo di Alta Specialità, Motta di Livenza
- **Roberto D'Amico**: Statistico, Università di Modena e Reggio Emilia
- **Luigi Tesio:** Fisiatra, Università di Milano, IRRCS Auxologico
- **Fabrizio Salvi:** Neurologo, Azienda USL di Bologna
- **Silvana Simi:** Rappresentante Pazienti, Gruppo Cochrane Internazionale Sclerosi Multipla, Milano

Steering Committee:

- **Paolo Boldrini:** Fisiatra, ULSS 9 Traviso – Ospedale Riabilitativo di Alta Specialità, Motta di Livenza
- **Antonio Bertolotto:** Neurologo, Azienda Ospedaliero-Universitaria San L.Gonzaga, Orbassano (TO)
- **Patrizia Cenni:** Neuroradiologo, Azienda USL di Ravenna, ospedale di Ravenna
- **Roberto Galeotti:** Radiologo Interventista, Azienda Ospedaliero universitaria di Ferrara
- **Roberto D'Alessandro:** Neurologo, Università di Bologna
- **Massimo Del Sette:** Neurologo, ASL 5, La Spezia
- **Graziella Filippini:** Neurologo – Metodologo, IRCCS "C. Besta, Milano/ Gruppo Cochrane Internazionale Sclerosi Multipla
- **Alessandro Liberati**: Epidemiologo-Metodologo, Università di Modena e Reggio Emilia/Assr – Regione Emilia Romagna, Bologna
- **Donato Papini:** Epidemiologo, Assr- Regione Emilia Romagna, Bologna
- **Fabrizio Salvi:** Neurologo, Azienda USL di Bologna
- **Silvana Simi:** Rappresentante Pazienti, Gruppo Cochrane Internazionale Sclerosi Multipla, Milano
- **Andrea Stella:** Chirurgo vascolare, Azienda Ospedaliero-Universitaria di Bologna
- **Luigi Tesio:** Fisiatra, Università di Milano, IRCCS Auxologico
- **Paolo Zamboni:** Chirurgo Vascolare, Azienda Ospedaliero universitaria di Ferrara
- **Roberto D'Amico:** Statistico, Università di Modena e Reggio Emilia

Accreditation Commission for Participating Centers:

- **Antonio Bertolotto:** Neurologo, Azienda Ospedaliero-Universitaria San L.Gonzaga, Orbassano (TO)
- **Patrizia Cenni:** Neuroradiologo , Azienda USL di Ravenna, ospedale di Ravenna
- **Massimo Del Sette:** Neurologo, ASL 5, La Spezia

- **Graziella Filippini:** Neurologo – Metodologo, IRCCS "C. Besta, Milano/ Gruppo Cochrane Internazionale Sclerosi Multipla
- **Roberto Galeotti:** Radiologo Interventista, Azienda Ospedaliero universitaria di Ferrara
- **Paolo Zamboni:** Chirurgo Vascolare, Azienda Ospedaliero universitaria di Ferrara

Center for Training and Coordinating of Functional Measures:

- **Luigi Tesio:** Fisiatra, Università di Milano, IRCCS Auxologico

Working Group on CRF and randomization:

- **Graziella Filippini:** Neurologo – Metodologo, IRCCS "C. Besta, Milano/ Gruppo Cochrane Internazionale Sclerosi Multipla
- **Stefania Galimberti:** Biostatistico Centro di Biostatistica per l'epidemiologia clinica, Università Milano-Bicocca Monza
- **CRO:** Medical Trials Analysis (MTA Italy)
- **Luigi Tesio:** Fisiatra, Università di Milano, IRCCS Auxologico
- **Grazia Valsecchi:** Biostatistico Centro di Biostatistica per l'epidemiologia clinica, Università Milano-Bicocca Monza

Center for MRI central reading:

- **Luca Massacesi**: Dipartimento di Neuroscienze, Azienda Ospedaliera Universitaria Careggi, Università di Firenze

Center for Echo-Doppler Training and Coordination:

- **Massimo Del Sette**: Neurologo, ASL 5, La Spezia
- **Paolo Zamboni:** Chirurgo Vascolare, Azienda Ospedaliero Universitaria di Ferrara

MRI Working Group:

- **Patrizia Cenni:** Neuroradiologo , Azienda USL di Ravenna, ospedale di Ravenna
- **Luca Massacesi,** Neurologo, Università di Firenze

Training Center for Venography and Angioplasty:

- **Patrizia Cenni:** Neuroradiologo , Azienda USL di Ravenna, ospedale di Ravenna
- **Roberto Galeotti:** Radiologo Interventista, Azienda Ospedaliero universitaria di Ferrara

Data Coordination Center (DCC):

- **D.ssa Valsecchi:** Centro di Biostatistica per l'epidemiologia clinica, Università Milano-Bicocca Monza
- **D.ssa Galimberti:** Centro di Biostatistica per l'epidemiologia clinica, Università Milano-Bicocca Monza

Independent Data Monitoring Committee (IDMC):

- **Paolo Bruzzi:** Epidemiologo-Metodologo, IRCCS Istiruto Nazionale per la Ricerca sul cancro, Genova
- **Gorge Ebers:** Action Research Professor of Clinical University of Oxford, Clinical Neurology University of Oxford (UK).
- **Luigi Pagliaro:** Professore Emerito di Medicina interna Università degli Studi di Palermo

Clinical Research Organization (CRO):

- Medical Trials Analysis (MTA Italy)

Studio CCSVI-SM: efficacia e sicurezza dell'angioplastica venosa nella SM

AGREEMENT FORM FOR CCSVI-MS PROTOCOL

As a participating physician, I declare to have read the Protocol approved by the Ethical Committee of the Azienda Ospedaliera Universitaria di Ferrara, regarding Venous angioplasty in multiple sclerosis

and to have discussed in full the objectives of the study and the protocol with the representative(s) of the Promoter.

I agree to participate in the study in accordance with the protocol and respecting the provisions of good clinical practice and the applicable local regulations.

I agree to keep reserved the contents of the protocol, all of the materials, and the unpublished information, documents and results, and not to divulge them to unauthorized persons.

I am aware that, if the Promoter should decide to interrupt the study prematurely, for whatever reason and at any time, I will receive a written notice from the same.

On the contrary, if I should decide to withdraw from the study, I will immediately inform the Promoter in writing.

Participating physician	
Name	
Signature	
Date	

Promoter's representative	*University Hospital Administration, Ferrara*
Name	*Professor Paolo Zamboni, M.D.*
Signature	
Date	

CONTENTS

1. Synopsis and study outline 7
2. Introduction 8
2.1. Multiple sclerosis: general considerations 8
2.2. CCSVI and the procedure for balloon dilatation of the extracranial veins 9
2.3. Study rationale 11
2.3.1. Physiopathologic rationale 11
2.3.2. Epidemiologic rationale 12
2.3.3. Clinical rationale 12
2.3.4. Public health and service organization rationale 12
3. Study objectives 12
4. Eligibility and exclusion criteria 13
4.1. Inclusion criteria 13
4.2. Exclusion criteria 13
4.3. Concomitant therapies 14
4.4. Criteria for echo-Doppler diagnosis of CCSVI 14
5. Recruitment and participating centers 14
6. Statistical plan and monitoring 15
6.1. Sample size and statistical power of the study 15
6.1.1. Patients with RRMS 16
6.1.2. Patients with SPMS 16
6.2. Monitoring modes of the study 17
6.3. Study monitoring and report 18
6.4. Statistical analysis 18
6.4.1. Relapsing Remitting MS patients 18
6.4.2. Secondary Progressive MS patients 19
6.5. Stopping rules 19
7. Procedure for randomization and data collection 20
7.1. Randomization 20
7.2. Data Collection 21
8. Blindness 21
9. Rules for conducting the study 21
10. Pre-randomization evaluations 22
10.1. Duties of the neurologist in charge 22
11. Echo-Doppler Ultrasonography 24
12. Venography and venous angioplastic procedure, standard and simulated 24
13. Post-treatment evaluation of patients 25
13.1. Endpoint and criteria for treatment efficacy 25
13.1.1. Functional clinical endpoint 26
13.1.2. RMI endpoint 27
13.2. Treatment safety and adverse events 27
13.3. Calendar of evaluations 28
13.3.1. Follow-up: programmed check-ups 28
13.3.2. Follow-up: unprogrammed check-ups 29
14. Rules for utilizing and publicizing data 29
Appendix 1. Protocol for ultrasonographic examinations 31
Appendix 2. Protocol for venography, angioplasty and simulated procedure 34
Appendix 3. Protocol for functional clinical evaluation and MRI 37
Appendix 4. Accreditation criteria for participating MD centers and quality control 48

14. Bibliography 51

1. SYNOPSIS AND STUDY OUTLINE

Controlled randomized clinical study to evaluate the clinical efficacy and safety of angioplastic surgery of the extracranial veins in multiple sclerosis patients, and diagnosis of chronic cerebrospinal venous insufficienza (CCSVI).
The study calls for a comparison of venography with venous angioplasty vs. a control treatment of venography only.
Eligible patients are aged 18-65 years and have multiple sclerosis defined according to McDonald's criteria and a confirmed diagnosis of CCSVI.
The study as two primary endpoints:

a) clinical (improvement/stability/worsening/fluctuating) in a concise functional endpoint at 12 months; and
b) magnetic resonance imaging (active lesions at 12 months).

The study is foreseen as being multi-centered, in keeping with the availability of centers having the clinical competence and equipment necessary for participation in the study (Appendice 4).
About 550 patients are expected to be recluted.
Follow-up is scheduled to last 15 months. Evaluations relative to the follow-up will be blind. Study flow can be seen in the diagram below.

Study flow diagram

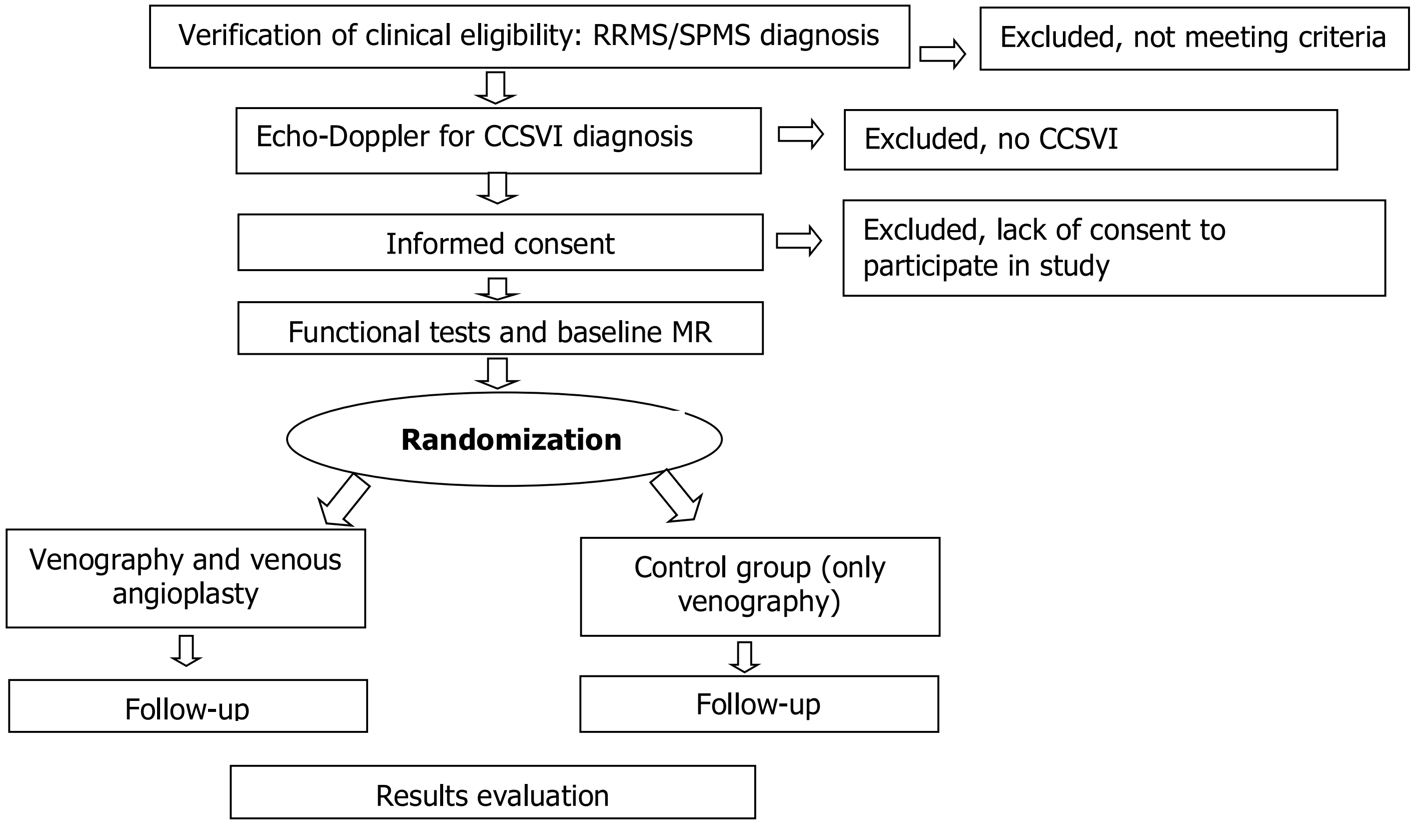

Table 1. Timing of examinations and tests

Time	Test
Pre-randomization	Distribution of information to patient
Ascertainments from protocol	Performance of ECD, blood tests
Base exam (maximum 15 days after pre-randomization)	Acquisition of valid consent*, Clinical exam, MR prescription
MRI (maximum 15 days before randomization)	MRI, basic functional tests
Randomization (0 time)	Randomization and angioplastic surgery (real or fictitious)
3 mesi	Clinical exam and functional tests
6 mesi	Clinical exam, ECD, MRI and functional tests
9 mesi	Clinical exam
12 mesi	Clinical exam, ECD, MRI and functional tests
12+3 mesi	Functional tests for confirmation

* consent must be acquired prior to MRI.

2. INTRODUCTION

2.1. Multiple sclerosis: general considerations

Multiple sclerosis (MS) is a chronic inflammatory demyelinating disease of the central nervous system (CNS) having a progressive course. It represents the second most common cause of disability in young adults (56). The incidence of this disease in the European population is 4-4.5 cases in 100,000, and the prevalence in the past 30 years has varied from 10 cases in 100,000 (Albania) to 188 cases in 100,000 (Finland), with an average of 83 cases in 100,000 (52). In the United States the prevalence of MS is 90 cases in 100,000 (31). The risk of developing the disease increases gradually as one goes from South to North for all biotypes, with Africans being less affected than Caucasians at all latitudes (38).

Females are more predisposed to MS than males (female/male ratio about 3:1). In about two-thirds of patients, MS onset is diagnosed between 20 and 40 years of age (52).

Based on the clinical course, MS is classified as (45):

- relapsing-remitting MS (RRMS): characterized by unpredictable exacerbations of the disease during which new symptoms may appear or already present symptoms become more severe. About 85% of MS patients exhibit this form in the first years.
- secondary-progressive MS (SPMS): a progressive phase follows the relapsing-remitting course in about 80% of patients within two decades (13), and few patients escape this evolution, which produces serious disability if the patient lives long enough.
- primary progressive MS (PPMS): characterized by a slow onset and a constant worsening of symptoms, without distinct attacks. It affects 10-15% of MS patients.
- progressive relapsing MS (PRMS): characterized by a progressive course with superimposed relapses.

The prognosis of MS varies according to the study and the inclusion criteria. In general the evolution of the disability is evaluated with changes in the Expanded Disability Statis Scale (EDSS) of Kurtzke. This scale is not linear and does not consider cognitive and sphincteral impairments that can be a primary component of the disability. In general a score of ***3-5*** indicates moderate disability, and a score of 6 or more indicates the loss of ability to walk without aid. In the Ontario cohort (14)(15)(20)(36)(37)(57)(78)(79)(80)(81) of more than 1,000 patients followed for over 20 years, after 5 years from onset, 30% of patients had an EDSS score of 2 or more, and 10% had a score of 5 or more. After 6-10 years, 30-40% of patients who started with RRMS had developed SPMS, with a progressive accumulation of disabilities. In the cohort of Olmstead County (USA), the time to reach an EDSS score of 6 was 24 years for the entire group, but 10 years for SPMS. Among the prevalent cases, independent of disease duration, 36% cannot walk without assistance or cannot walk at all, and 14% are either confined to a wheelchair (50) or bedridden. However, these studies are based on diagnoses of MS defined by Poser's criteria. Therefore, the use of different diagnostic criteria based on MRI, which allow for an earlier diagnosis and possibly in milder cases, could modify the prognostic profile of the disease. In any case, MS produces serious disability, over a long period, in more than half of patients, and at least moderate disabilty in the remaining 25%.

There are currently many pharmacological treatments available for MS in the RRMS form, while only one treatment (interferon β-1b) is approved in Europe (but not by the FDA) for the SPMS form (76).

First-line treatments for the RRMS form are interferon β and glatiramer acetate. The effect of such treatments is a moderate reduction in the risk of relapses and the progression of the disease at the 2-year follow-up (54). There are no experimental or clinical verifications, statistically or epidemiologically validated, that the course of the disease is substantially modified in the long term, even if recent observational studies seem to indicate a possible slowing of disease progression (71). These treatments have significant side effects on the patients' quality of life, such as flu-like symptoms, headache, lymphopenia, liver pain ("risk" is not a side effect), depression and fatigue from interferon, and local reactions at the injection site for glatiramer.

Second-line treatments, reserved for patients whose disease is not controllable with immunomodulator drugs (interferon and glatiramer) are mitossantrone and natalizumab.

Mitossantrone reduces the frequency of relapses and the number of active lesions observed by MRI but causes serious side effects on the heart and the haemapoietic system (cardiotoxicity and myeloid leukemia in the years following treatment).

Natalizumab has been shown to be consistently efficacious (42%) in reducing the risk of disease progression after two years of treatment, but increases the risk (not yet determined precisely but definitely superior to 1 in 1,000 after two years of treatment) of multifocal progressive leukoencephalopathy.

In conclusion, there is currently no pharmacological treatment available, having a good profile of safety and tolerability, that is able to change, surely and significantly, the natural course of MS. New pharmalogical treatments in the process of approval seem to be more efficacious than interferon and glatiramer in reducing the frequency of relapses and the increase in disabilities, but their safety profile has not yet been well established.

2.2. CCSVI and the procedure for balloon dilatation of the extracranial veins

Recently there have been reports of observations of the possible presence of anomalies in the venous circle in patients with MS (89)(92), anomalies absent in normal controls or in other neurological diseases (OND). These reports have aroused great interest, both in the scientific world and, above all, in the patient communities of associations whose purpose is to help affected persons and to promote scientific research on MS.

Currently results are available of some studies conducted in Italy, the United States, Germany, Poland, and Jordan.

Zamboni and colleagues at the University of Ferrara, Italy, examined 65 patients with MS and 235 controls who were either normal or affected by OND (89), and successively 109 MS patients were compared to 177 normal or OND controls (92). These cases were studied by means of extracraial and transcranial echo-colour Doppler to evaluate, in particular, five parameters:

a) reflux in the jugular and/or vertebral veins; b) reflux in the deep intracerebral veins; c) detection, using the echo-Doppler B-mode, of stenoses proximal to the jugular vein; d) absence of reflux, evaluated by echo-Doppler, of the jugular vein and/or vertebral veins; and e) negative difference in the cross-sectional area (ΔCSA) evaluated in the jugular vein, by subtracting the values measured in the supine position from those measured in the erect position.

Without previous validating studies, the Authors have defined as pathological at least two of the above alterations and have called this condition "chronic cerebrospinal venous insufficiency" (CCSVI). In 100% of the MS cases examined by Zamboni and colleagues, at least two of these alterations were found, as compared to 0% among normal controls and patients affected by OND (89). In addition, a relationship was observed between the clinical course and the pattern of venous anomaly, for example, anomalies of the azygous vein were more frequent in the primary progressive form (PPMS), where involvement of the spinal cord is more frequent and more serious. The 65 patients and 48 of the controls underwent venography by means of catheterization, and stenosis of the jugular or azygous vein was found in 91% and 86%, respectively, of the MS patients (89). The same Authors have reported the results of an open study of 65 patients with MS (35 RRMS, 20 SPMS, and 10 PPMS) who underwent angioplasty for vein dilatation (88).

A second group of researchers directed by Zivadinov of the University di Buffalo (NY, USA) examined the venous system of 500 subjects, including 160 healthy controls, 280 MS patients (mostly RRMS), and 60 patients with OND. They discovered that 56% of those with MS and 22% of the healthy controls suffered a restriction of the extracranial veins. Moreover, the frequency of venous anomalies was greater in patients with more serious MS (95).

At the University Hospital Charité (Humboldt University, Berlin), 56 pazienti (41 RRMS, 15 SPMS) and 20 healthy controls underwent echo-colour Doppler of the internal jugular veins (IJVs), vertebral veins (VVs), and the intracranial veins. The diagnostic criteria for CCSVI of Zamboni and colleagues were applied. Blood flow direction in the IJVs and VVs was normal in all subjects, and no stenoses of the IJV were found. Flow volume in the IJVs and the VVs showed no differences between MS patients and healthy controls in the supine position. However, changing to the erect position increased flow volume among the patients (318 ml/min±242 vs 123±109 ml/min; $p<0.001$). No differences were observed between patients and controls for the intracranial veins or during the Valsalva manoeuvre. None of the patients was diagnosed with CCSVI according to the adopted criteria. These Authors assert that there is no evidence to sustain the hypothesis that the presence of anomalies in the venous circle plays a role in MS pathogenesis. They suggest the need for further studies to explain the difference between patients and controls in the regulation of venous flow volume (19).

The results of another study, conducted in Germany at the Ruhr-Universität Bochum (38), have recently been published. This study included 10 MS patients (2 RRMS, 7 SPMS, 1 PPMS) and 7 controls (2 healthy, 1 with global transitory amnesia, 2 with migraine, and 2 with dysaesthesic syndromes). Diagnosis of CCSVI was made in 2 of the 10 MS patients and in none of the controls. The Authors concluded that the pathogenetic importance of CCSVI for MS was not demonstrated.

At the private hospital SANA (Pszczyna, Poland), an uncontrolled study was conducted using echo-Doppler of the extracranial veins of 70 patient (49 RRMS, 16 SPMS, 5 PPMS). Diagnosis of CCSVI

was made for 63 (90%) of the patients, and the most frequently found anomalies were inversed direction of a valve and the presence of septums at the juncture between the jugular and anonymous veins. The Authors concluded that MS is significantly associated with CCSVI (59).

At King Abdallah University Hospital in Jordan, diagnosis of CCSVI was made in 21 of 25 patients examined by echo-colour Doppler to study the internal jugular veins; none of the 25 controls was thus diagnosed. The conclusion drawn was that haemodynamic and morphologic alterations of the internal jugular veins are significantly associated with MS (5).

In summary, the study results available in the literature are conflicting, with a prevalence of CCSVI in MS patients ranging from 0-100%. These differences can be attributed to various factors, including the low quality of some of the studies, in particular the absence of blindness on the part of the experimenters in the majority of cases, the methodology adopted, the varying accuracy of the diagnostic tests (including the apparatus used and the examiners' experience) and the study setting.

Therefore, additional studies having better methodologies are needed to test the hypothesis of an association between CCSVI and MS.

2.3. Study rationale

2.3.1. Physiopathologic rationale

Since the mid-1800s, neuropathologic studies of MS have emphasized a perivenular distribution of the inflammatory infiltrates and a close resemblance to the neuropathologic picture of the plaques of demyelinization.

According to the hypothesis of Zamboni and colleagues, as reported above, alterations in the veins of the neck, probably due to malformations, cause changes in venous flow with inversion of flow in the intracerebral veins, an anomalous venous drainage, iron deposition in perivascular and intraparenchymal sites (86), alterations in the haemato-encephalic barrier, and the beginning of a cascade of inflammatory events that can persist over time, even through autoimmune-type mechanisms.

Previous research has shown the autoimmune origin of the disease (12), from the animal model to pathological findings, to the partial response to immunomodulating and immunosuppressive therapies.

Epidemiological studies and studies of monozygote twins, with both or only one affected by MS, have shown that, in addition to factors of genetic predisposition, which affect about 15-20% of cases, there exist external and environmental factors, not yet identified, that provoke the disease in 60-70% of cases.

The neuropathology involves a process of demyelinization and axonal damage, both primary and as a result of inflammation (23), but shows no signs of venous stasis or haemorrhage.

It should be noted that Zamboni's data have aroused interest because they suggest a sequence of coherent pathogenetic events. In fact, stenoses of the exracranial veins, congenital and genetically determined, can bring about an inversion of venous flow, with the following consequences: anomalous activation of the endothelial cells, opening of the haemato-encephalic barrier, entering into the central nervous system of lymphomonocytes that come into contact with antigens that were previously sequestered, and thus the beginning of an autoimmune cascade that lasts over time. From this point of view, repairing the stenosis and re-establishing a correct venous flow from the encephalus towards the heart could have therapeutic effects, especially if the procedure were performed precociously. In effect, Zamboni and colleagues have treated 65 cases of MS with angioplastic techniques (88) with follow-up for 18 months, reporting clinical improvements above

all in relapsing-remitting (RRMS) forms, but also finding a high degree of restenosis in the jugular veins. This open study cannot necessarily lead to the conclusion of even partial efficacy of the therapy, but it demonstrates that angioplasty is capable of producing positive effects, even if considerable problems exist regarding the possibility of restenosis. The Authors are aware of the limitations of the data and sustain the need for a controlled randomized study.

Numerous objections have been raised regarding Zamboni's hypotheses, and epidemiological studies are currently underway to evaluate, using the same techniques as those utilized by Zamboni (89), the prevalence of venous anomalies in MS patients, those with other diseases and healthy controls.

2.3.2. Epidemiologic rationale

Multiple sclerosis is characterized by a great variability in clinical course, both among different patients and also in the same patient in different periods. For this reason it is very difficult to draw conclusions about the efficacy of a treatment with observational studies within an acceptable time. In fact, such studies should be conducted on a large number of patients for many years in order to observe any substantial differences with respect to the disease prognosis observable in historic cohorts. Therefore, the only instrument that can lead to a conclusion about the efficacy of a treatment within a reasonable time is a controlled randomized clinical study based essentially on clinical outcomes, changes in which are observable over a short period.

2.3.3. Clinical rationale

The data resulting from the only treatment study published to date (88) are very limited because the study population was small, the study was conducted in an open form, and effects of the treatment were measured partially and without a rigorous time-table. These limitations indicate the need for a controlled clinical study capable of providing an answer, not only about the efficacy of venous angioplasty on the inflammatory component, but also about many symptoms, such as fatigue, motorial deficit and pain, which characterize the progressive phase of the disease. In fact, an important part of patients' expectations, sustained and amplified by anecdotal data and by an improvement in the quality of life reported in the Zamboni study (88), has to do precisely with these aspects.

2.3.4. Public health and service organization rationale

As stated above, the pharmacological treatments currently available for MS therapy can modify, to a limited degree and over the long term, the natural evolution of the disease. However, they cause numerous side effects and are, at the moment, not easily proposable for the duration of the disease, which corresponds to the rest of the patient's life. Currently there are no elements for evaluating whether new medicines that are in the approval process or undergoing experimentation will be able to alter the natural course of MS. Thus it is perfectly understandable that a nonpharmalogical treatment which hypothesizes an improvement in the clinical picture of the disease, that is, the intervention proposed by Zamboni, arouses high hopes for a confirmation of the scientific validity of the treatment.

3. STUDY OBJECTIVES

The main objective of this study is to evaluate the efficacy and safety of dilatation of the extracranial veins, by means of angioplasty, in patients with relapsing-remitting multiple sclerosis (RRMS) or secondary progressive multiple sclerosis (SPMS) and with CCSVI as defined by the inclusion criteria.

Primary objectives at 12 months: (appendix 3)

- clinical response measured by a "concise functional endpoint" obtained by means of the integration of functional indicators, measured with an instrumental method and clinically significant, related to walking, balance, manual dexterity, sphincteral control, and visual acuity
- disease activity measured by magnetic resonance imaging (MRI)

Sccondary objectives at 12 months: (appendix 3)

- variation in disability measured by points on the EDSS and PASAT
- proportion of patients with disease relapses measured by a categorical endpoint (0, 1-2, >2 relapses)
- percentage of patients with restenosis in the treated arm of the study
- stratification of the primary and secondary objectives in study arms with and without restenosis
- fatigue measured with specific indicators
- emotional state
- cognitive state
- impact of bladder incontinence
- adverse events correlated with the treatment

Another secondary objective is to calculate the proportion of patients with CCSVI confirmed by venography.

4. ELIGIBILITY AND EXCLUSION CRITERIA

4.1. Inclusion criteria

1) Age 18-65 years
2) MS defined according to McDonald's criteria (2005) with relapsing-remitting (RRMS) or secondary progressive (SPMS) course
3) CCSVI diagnosed with echo-Doppler according to the criteria specified in point 4.3
4) EDSS from 2 to 5.5
5) Disease duration from diagnosis to evaluation <10 years
6) Stable neurological condition without relapse for at least 30 days
7) Patient not undergoing therapy, or undergoing immunomodulating or immunosuppressive therapy without modification for at least 6 months

Included patients must not start or modify immunomodulating or immunosuppressive therapy during the study.
As regards the use of "symptomatic" therapies, better defined here as symptomatic drugs, see appendix 3, point "Criteria for potential postponement or inadmissibility of functional evaluations."
In any event, all pharmalogical and rehabilitative treatments (motorial re-education and balance, walking,_and cognitive training), carried out during the study and in the six months previous, are to be recorded in the clinical record file **(CRF).**

4.2. Exclusion criteria

1. Patients who have previously undergone a venous angioplastic intervention

2. Patients undergoing natalizumab (Tysabri) or fingolimod therapy
3. Patients treated with botulinic toxin in the 3 months preceding the start of the study
4. Patients with implanted infusion or neurostimulator pumps in the 3 months preceding the start of the study
5. Participation in another clinical trial during the course of the study
6. Use of experimental drugs or participation in another clinical trial in the 3 months prior to the screening examination
7. Current or previous use of these experimental drugs: cladribine and/or laquinimod
8. Current or previous radiotherapy performed for any reason
9. Pregnancy or desired pregnancy or refusal of contraceptive methods
10. Contraindications upon venography:
    - known/documented *nota* hereditary thrombophilia
    - previous adverse reactions after administration of iodized contrast mediums or presence of patological conditions that could favor the insurgence of collateral reactions to the introduction of contrast mediums: severe renal insufficiency, severe hepatic insufficiency, severe cardiac insufficiency, Walderstrom's paraproteinaemia, or multiple myeloma.
11. Contraindications for MRI and/or the contrast medium for performing MRI
12. Patients unable to perform one or more basal functional tests for any impairment or motivation (e.g., blindness, refusal to walk without aid, etc.)
13. Concomitant or previous pathologies that could cause changes in motor and/or vision and/or sphincteral and/or cognitive functions (see appendix 3, "Criteria for potential postponement or inadmissibility of functional evaluations").
14. Patients unable to provide valid consent.
15. Patients unable to carry out the program of observation and control following treatment
16. Patients with known/documented congenital, ischaemic, or arrhythmic cardiopathies.

4.3. Concomitant therapies

If not explicitly mentioned in the inclusion or exclusion criteria or in appendix 4, the concomitant therapies for chronic pathologies not correlated with MS should be continued throughout the study period. All of the drugs assumed during the study should be recorded in the data collection file of the study.

4.4. Criteria for echo-Doppler diagnosis of CCSVI

A diagnosis of CCSVI requires the presence of at least 2 of the following 5 criteria:

1. Reflux constantly present in internal jugular and/or vertebral veins (patient in both examination positions: sitting and supine.
2. Reflux in the intracranial veins (Rosenthal's vein, transverse sinus, Petrosus sinuses, cavernous sinus).
3. Presence of anatomic alterations (septums, valvular malformations, double canal, CSA <0.3 cm2).
4. Absence of flow in IJV and/or VV after repeated deep inhalations with the patient in both examination positions (sitting and supine). The finding of absence of flow in only one body position becomes a useful criterion even if flow is found in the other position.
5. CSA of the IJV in sitting > CSA of the IJV in supine.

For a detailed description of the neurosonologic diagnosis of CCSVI, see appendix 1.

5. RECRUITMENT AND PARTICIPATING CENTERS

The multi-centered study involves MS centers that can coordinate and perform correctly the pre-randomization procedures, the diagnostic and venous angioplasty procedures, and the follow-up exams.

The participating centers are those that present internal MS Units with at least 400 patients under their care. Each center should contribute between 30 and 50 patients to the study. The training and recruitment of patients for the study are two separate functions carried out by different operating groups.

All of the participating centers must be accredited by the national healthcare system (SSN).

The participation of MS centers and their associated angiographic and sonologic centers is approved by the ethical committee .
The specific requisites for center accreditation are described in appendix 4.

6. STATISTICAL PLAN AND MONITORING

6.1 Sample size and statistical power of the study

The proponent group has agreed on the fact that the endpoints currently used in clinical pharmalogical studies are the object of much criticism, on both clinical and methodological levels (10)(20).

For this reason the proponent group convened to identify two primary endpoints:

1. the classification of each patient as improved/ stable/worsened/fluctuating on the basis of a series of functional parameters relative to specific impairments present in different measures in patients with RRMS and SPMS; and

2. magnetic resonance imaging (MRI).

Two secondary endpoints were then defined, both being of a functional nature (EDSS and PASAT).

It must be remembered that the study was designed taking into account: a) the need to include all of the categories of patients who are potential candidates for finding, through angioplastic intervention, a possible therapeutic response; and b) the need for a short enlistment time for the case registry and a follow-up period of not more than 15 months.
Since the two diagnostic categories (RRMS and SPMS) will have different numbers and characteristics of response, the estimates proposed below imply that the study will have a statistical power that is *a priori* different for the two groups. It is also probable that, as described in more detail below, the study will produce stronger verifiable results in the first subgroup than in the second. For the patients with SPMS, the study will represent a first step that will necessitate confirmation, unless, obviously, it is decided to prolong the periods for recruitment and observation during the first approval phase of the protocol.

As stated, the study calls for the enlistment of two types of patients:
1. patients with relapsing-remitting multiple sclerosis (RRMS); and
2. patients with secondary progressive multiple sclerosis (SPMS).

The estimate of the sample size and the analyses have been diversified for the two groups.

6.1.1 Patients with RRMS

The sample size was estimated on the basis of one of the two principal endpoints, MRI. The selected outcome measure was the number of active lesions, defined as those that absorb the contrast medium, the new T2 positive lesions, and the T2 positive lesions that increase in size. It is hypothesized that in the control group, the average number of active lesions is equal to 6 with a standard deviation of $\pm$7.6. The enlistment of 357 patients allocated into two arms according to a randomization ratio of 1:2 (119 in the control arm and 238 in the experimental arm) will allow for, with an alpha error of 0.05 and a power of 80%, an absolute reduction of 2.4 in lesion average, or rather a variation from 6 lesions in the control arm to 3.6 in the experimental arm. That great a difference corresponds to a relative reduction of 40%.
This sample size is also considered sufficient for evaluating the efficacy of the angioplastic treatment with respect to the clinical indicator.
The estimated sample size with respect to the MRI endpoint is sufficient to obtain statistically significant differences (α= 0.05) even for the secondary PASAT endpoint (cap. 3). In fact, if it is hypothesized that in the experimental arm the average PASAT value is 0.50, and that in the control arm it is, at the most, equal to 0.25 (SD=$\pm$**0.5**) (estimated from individual differences calculated by comparing observed values after one year with those obtained at the start / with basal values), a sample of 357 patients (238 experimental, 119 control) is sufficient to obtain such a difference (0.50-0.25=0.25) with a statistical power of 99%.

6.1.2 Patients with SPMS

Current knowledge about the natural history of SPMS, the efficacy of available treatments and, as stated, the "pragmatic" nature of the selected experimental design, render impossible an accurate estimation of the sample size necessary to reasonably expect to have, at the end of the study, a predefined statistical power.
The relatively short duration of follow-up (15 months) has induced the choice of a clinical endpoint different from that traditionally used for longer follow-ups. This endpoint is derived from the integrated evaluation of five clinical parameters for which the specific psychometric characteristics are known (see paragraph 12.1.1). However, the proposed decisional algorithm, which integrates the parameters in order to classify each individual case, has not yet been validated clinically. This lack of validation does not allow for an accurate estimate of the sample size at the moment the study begins and, for this reason, a pragmatic criterion has been selected on the basis of the recruitment capacity of the centers involved in the study.
On the hypothesis that 10 centers will be able to participate in the study, about 210 patients with SPMS should be recruited during the enlistment period.
These patients will also be allocated into two arms according to a randomization ratio of 2:1, or 140 in the treatment arm and 70 in the control arm. This numerous sampling, merely by way of example, could allow for attaining a 93% statistical power with an alpha error of 5% of the clinically significant differences in the variations of the proportions of patients who worsened in the two groups by comparing each of the five clinical parameters evaluated.
Table 2 reports the details of the hypotheses formulated on the basis of data available in the scientific literature (7)(8)(66)(83).

Table 2. Hypothesis for calculating the statistical power.

Parameters	Control			Treatment			Power
	%with impairments at baseline	% at 1 year	Increase in% worsened	%with impairments at baseline	% at 1 year	Increase in% worsened	Control N=70 Treatment N=140
Visual	25%	40%	+15%	25%	26%	+1%	0.93

acuity							
Sphincteral control	25%	40%	+15%	25%	26%	+1%	0.93
Manual dexterity	20%	35%	+15%	20%	26%	+1%	0.93
Balance	70%	85%	+15%	70%	71%	+1%	0.93
Walking	40%	55%	+15%	40%	26%	+1%	0.93

Therefore, it is reasonable that the results derived from the study of SPMS patients be considered preliminary, unless the Independent Data Monitoring Committee (IDMC) proposes to the Steering Committee that the recruitment period be prolonged and the follow-up period last at least two years. It should be remembered that in the United States register www.clinicaltrial.gov (trial code NCT01089686), an American study having the same objective is recorded. In perspective, this could allow for international collaboration on a cumulative data analysis (perspective objective analysis) relative to the common endpoint of the two studies.
The estimated sample size for evaluating treatment efficacy in SPMS patients is such as to obtain statistically significant differences ($\alpha = 0.05$) even for the secondary PASAT endpoint (cap. 3). On the hypothesis that the average PASAT value (estimated from the calculated individual differences comparing the values observed at one year with those obtained at the start) in the control arm is at least equal to 0.10 (sd=$\pm$0.35), while in the treatment arm it is 0.30, a sample of 210 patients (140 treatment, 70 control) is sufficient to obtain a difference of 0.20 (0.30-0.10) with an alpha error of 0.05 and a statistical power of 97%.

6.2. Monitoring modes for the study

The monitoring procedures will be entrusted to a Contract Research Organization (CRO). During the study, monitoring personnel will visit the participating centers regularly to verify the completeness of the data, the accuracy of completion of the data collection forms, adherence to the protocol and techniques of good clinical practice (GCP), the state of enlistments and any problems.
The clinical investigator of the participating center should be available to assist the monitor during these visits. The CRO will also provide active monitoring with respect to the follow-up criteria on the part of the participating centers.
The investigator should provide the monitor with access to clinical data and to data regarding hospitalization to confirm the consistency of the information recorded on the data collection form. All information relative to patient identity must be reserved according to law (D.Lvo '96/2003) at the participating center. Standard monitoring procedures call for a complete verification of the validity of adhesion to the study, adherence to the inclusion/exclusion criteria, documentation of serious adverse events, and the recording of variables in safety and efficacy.

The patients' personal and sensitive information will be made anonymous, and study subjects will be identified on the data collection form only by a code that is common to all of the participating centers. If, for exceptional security reasons or upon the request of legal authorities, the need to identify a patient should arise, both the Data Coordinating Center and the investigator will be bound to treat this information in a reserved manner according to the previsions of the law in force.
The investigators will have to file the complete documentation of the patients under study, the medical and demographic information, and a copy of the informed consent signed by the patient.
All of the information reported on the data collection form will have to be traceable to the original clinical documentation contained in the patient's clinical file.
The study documents will have to be filed by the experimenters for a period of seven years according to Article 18, paragraph 1 of the D.l. 200 dated 6 November 2007.

6.3. Study monitoring and report

The scientific responsibility for monitoring the course of the study will be entrusted to an Independent Data Monitoring Committee (IDMC) that will work in close contact with the Data Coordination Center (DCC) and the CRO.
As regards toxicity, the IDMC will convene every two months during the first year, and every four months beginning with the second year, to evaluate the state of the treatment groups and to inform the steering committee of any decisions to be made for an early interruption of the study.
The study will be interrupted if the frequency of serious adverse events is greater than 2%.
During the course of the study, the IDMC will also evaluate, in collaboration with the Steering Committee, the publication of results of any concomitant studies, so as to consider a possible early interruption of the study.
Statistical analysis will be conducted without opening the masking/ unmasking. In order to ensure that the "stopping rules" are respected, usually the independent monitoring committee has open access to the data during the course of the study.

The statistical analysis will be conducted without the opening of the masking. In order to be able to comply with the "stopping rules" only the Independent Monitoring Committee has access to open data while conducting the study.

6.4. Statistical analysis

The statistical analysis will be the responsibility of the CCD and will be conducted at the end of the study without the opening of the masking.
The main analysis will be conducted according to the Intention To Treat (ITT) principle, separately on the two subpopulations (SMRR and SMSP), and this results in the fact that all randomized patients (after sonoscopic diagnosis of CCSVI) will be considered in the group of assigned treatment. A secondary analysis will be "Per Protocol" (PP) and will therefore consider all randomized patients (after sonoscopic diagnosis of CCSVI) that have been positive (true CCSVI) also for venography. Positive false patients are expected to be up to 10%. The holding of randomization will be monitored by post-analysis of the percentage of negative venographies in treaties and controls. Patients with no 1-year endpoint (defined as drop-outs) will be excluded from both analyzes. It is expected that there will be at most 5% of unmanageable patients. Sensitivity analysis will be performed that will consider different endpoint values for such patients.
A comprehensive analysis (both ITT and PP) will be performed on the entire sample in the study (SMRR + SMSP).

6.4.1 Relapsing Remitting MS patients

In this group of patients, the analysis of the treatment effect on the primary endpoint (mean number of 1-year RM lesions) will be performed using a probability-based test (LRT) assuming a Negative Binomial Model (two-tier test , α = 5%). This comparison will also be evaluated by adjusting for some relevant factors such as time from disease diagnosis, concomitant treatments, and EDSS recruitment.
A descriptive analysis of the components of the primary endpoint will also be conducted, taking into consideration the effect of the treatment on the T1 and T2 positive lesions separately.
The effect of the treatment will also be evaluated on the clinical endpoint by comparing the proportions of subjects classified as "Improved" compared to the combined clinical endpoint as defined in section 13.1.1 (two-tier test on proportions, α = 5%).

The one-year relapse rate (secondary endpoint) in the two treatment groups will be compared on the basis of a probability-based test assuming a Poisson model (also in the regression version adjusted for the same relevant factors considered in the endpoint analysis primary). The appropriateness of the Poisson model to the study data will be evaluated, and if the assumption of variance does not hold (overdispersion) will be considered as alternative models that will overshadow this condition (ie, mixed-pattern poisson model).

6.4.2 Secondary Progressive MS patients

For the subpopulation of patients with SMSP, the primary analysis on the effect of treatment will be performed with a test for the comparison of the proportion of subjects in the two groups, which are classified as "Improved" compared to the combined clinical endpoint the definition given in section 13.1.1 (two-tier test, $\alpha = 5\%$). An analysis will also be carried out that takes into account some relevant factors such as sex, time from disease diagnosis, concomitant treatment, EDSS recruitment, using the logistic regression model.
The following secondary analysis of the effect of the treatment will also be carried out:
1) analysis of association between treatment and distribution of individual items of combined endpoint (see section 12.1.1);
2) Effect analysis on the single functional endpoint of the combined endpoint, using the Holzberg correction method for multiplicity of tests (12).
The effect of the treatment will also be evaluated on the magnetic resonance endpoint by comparing the average number of lesions to 1 year (two-tailed LRT test for mean comparison by assuming a Binomial Negative model, $\alpha = 5\%$).
Both subpopulations will be analyzed for all the secondary endpoints listed in section 3 and not specifically mentioned.
Where parametric methods are used, a sensitivity analysis will be performed based on non-parametric methods as well. Any change from the planned statistical methods will be appropriately documented.
It should be remembered that in the US register www.clinicaltrial.gov (trial with NCT01089686) an American study has been recorded that evaluates the same objective. This could allow for international collaboration for a cumulative data analysis (prospective metaanalysis) for the common endpoints of the two studies. In addition, at the time of the final drafting of this protocol, contacts were established with a group of Canadian researchers (coordinated by Prof. David Sackett and Andreas Laupacis - Mac Master University, Ontario) who are involved in the design of a randomized controlled trial that has many analogies this protocol. The currently available information does not allow us to assess whether and how any prospective data combination will be possible. The Steering Committee, however, is committed to maintaining frequent contacts with this research group in order to allow a mutual information advantage for the two researches.

6.5. Stopping rules

Guidelines have been built to ensure that the study is discontinued as soon as possible, if its application should be associated with the occurrence of an unacceptable proportion of serious adverse events.
The applied method follows a Bayesian approach (21) (23) and the following table shows experimental results that provide a 90% or more a-posterior probability of obtaining a percentage of serious adverse events greater than the maximum acceptable (pmax), fixed to 4%. These values indicate the minimum number of adverse events for the number of patients enrolled, which should alert the IDMC to the possibility of anticipating a premature stop of the study.

It is assumed that the number of events follows a Binomial distribution and that the a priori probability distribution of the event is a Beta (1,1), which corresponds to a non-informative distribution Uniform.

N. eventi avversi seri	N. cumulativo di pazienti
2	13-27
3	28-44
4	45-61
5	62-79
6	80-98
7	99-117
8	118-137
9	138-157
10	158-177
11	178-198
12	199-218
13	219-239
14	240-260
15	261-281
16	282-302
17	303-323
18	324-345
19	346-366
20	367-388
21	389-410
22	411-431
23	432-453
24	454-475
25	476-497
26	498-519
27	520-541
28	542-563
29	564-585
30	586-608
31	609-630
32	631-652
33	653-674
34	675-679

7. PROCEDURE FOR RANDOMIZATION AND DATA COLLECTION

7.1. Randomization

Responsibility for organizing the modes of computerized randomization *randomizzazione informatizzata* (including all of the necessary procedures) and their practical execution will be entrusted to an independent center, the Data Coordination Center (DCC), different from the one that processes the criteria and performance parameters of the study.

This center will be responsible for the electronic data filing and for maintaining the necessary contacts with the participating subgroups as well as with the Steering Committee, the Data Monitoring Committe and the CRO.

The center will also be in charge of scheduling *predisposizione* the electronic **CRF (e-CRF**); data access will be protected by user name and password to which only the director of the randomization center and the center's director of computer infrastructure will have access.

All of the requested information will have to be recorded in the data collection file in electronic form. Monitors will have to check the completeness and accuracy of the collected data and instruct the personnel of the participating centers to make every correction or change requested (query). An electronic query system, managed by the monitor, will be available, and every correction of original data will have to be approved by the monitor. The traceability of all data entered into the system will be guaranteed.
Randomization will be stratified by participating center and disease course, RRMS or SPMS.

7.2. Data Collection

The CRO will be responsible for the electronic storage of data and the maintenance of necessary contacts with the centers participating in the study as well as with the Steering Committee, CCD, and IDMC.
The predisposition of electronic e-CRF (e-CRF) will be based on e-CRF structured by the e-CRF Working Group and the implementation of the web data collection system will be entrusted to the CRO.
Data access will be protected by username and password according to different user profiles. In particular, the centers will only have access to their center data, the IT infrastructure manager of the CRO and the data quality manager in the CCD will have access to the entire database.
The Data Quality Manager in the CCD may download the format in agreement with the CRO and at any time the data periodically present in the current database for quality audits. Each access will be traced.
The screening card, graphic-computer documentation and examination reports, baseline data and all clinical documentation must be included in the electronic data collection (e-CRF) data sheet and stored in paper format by the PI of the respective centre. The CRO monitors will have to check the completeness and accuracy of the collected data and educate the staff of the participating centers to the study to make any corrections or modifications required (queries). An electronic query system managed by the monitor will be available and any correction of the original data will have to be approved by the responsible physician. It will ensure the traceability of all the data (original and correct) inserted in the system.

8. BLINDNESS

With respect to assignment to treatment group, the following will be blinded:

- the patient
- the neurologist in charge
- the investigator who will measure the primary and secondary clinical endpoints
- the Clinical Endpoint Commission
- the neuroradiologist responsible for centralized measurement of the RMI endpoint (Center for Centralized MRI Reading)
- the Data Analysis Center, to be distinguished from the Randomization Center of the study
  N.B: The physician and angiography personnel who will perform the venography and venous angioplasty (standard or simulated) will be aware of the treatment carried out. Heparin or a placebo, indistinguishable from heparin, will be prescribed to the patient by the physician-angiographist and delivered directly to the patient by the personnel present in the angiography room.

9. RULES FOR CONDUCTING THE STUDY

Ethics and Good Clinical Practice

This study will be conducted in accordance with the rules of good clinical practice (GCP) and the laws in force:

- DM 15 July 1997 no. 162: Adoption *Recepimento* of guidelines of the European Union for Good Clinical Practice for performing clinical experiments on medicines.
- DL 24 June 2003 no. 211: Taking effect of directive 2001/20/CE relative to the application of good clinical practice for performing clinical experiments on medicines for clinical use.
- DM 17 December 2004: Prescriptions and conditions of a general character relative to performing clinical experiments on medicines, with particular reference to those intended to improve clinical practice, an integral part of health care assistance. *quale parte integrante dell'assistenza sanitaria.*
- D.Lvo 196/2003 "Code on the subject of protection of personal data."

The experimenters, by signing the protocol, underwrite their adherence to the instructions and procedures described therein, declaring their own adherence to the principles of GCP and avoiding any conflicts of interest. The groups constituting the drafting committee for designing the protocol under examination will be distinguished from those who evaluate the venographic and angioplastic post-treatment effects.

Amendments to the protocol

Any changes to protocol will be made in the form of an amendment proposed to the Executive Committee (EC) of the Coordination and Promotion Center and evaluated by that person.

Changes in study management

No changes to the study are permitted unless a relative amendment to the protocol has been previously adopted. Any deviation from the protocol must be documented in the clinical file.

10. PRE-RANDOMIZATION EVALUATIONS

The pre-randomization evaluation process is coordinated by the neurologist who directs the study (neurologist in charge) at each participating MS center, with the objective of verifying that the patient respects the inclusion criteria called for in the protocol.

10.1. Duties of the neurologist in charge

For all of the eligible patients the neurologist in charge will record in the CRF:

- verification of RRMS/SPMS diagnosis
- detailed history of the disease, listing the single attacks episodes?
- previous therapies of the immunomodulator or immunosuppressive type and the relative starting and suspension dates
- any concomitant pathologies and related therapies
- neurological examination with EDSS score
- distribution of useful technical information, delivery of information and consent forms
- agreed-upon scheduling of the neurological examination for inclusion and of EDSS re-evaluation and formal acquisition of valid consent
- agreed-upon scheduling of the echo-Doppler examination
- results of the echo-Doppler examination

For the included patients the neurologist in charge:

- arranges an appointment for performance of the basal functional tests

- arranges an appointment for performance of the basal MRI test
- carries out the neurological examination, assigning a basal EDSS score
- completes the randomization form and sends it to the centralized unit for randomization
- arranges an appointment for extracranial venous angioplasty
- organizes the successive physical exams and tests according to the protocol and gives the patient a personal flow-chart on which are listed the dates and scheduled exams and tests

Eligible patients will follow this evaluative procedure:

a) Pre-randomization meeting

On occasion of the pre-randomization meeting, the neurologist in charge shows the patient the aims and the procedures of the study and gives him or her a copy of the information sheet and the consent form for participation. In particular, the neurologist informs the patient concerning the characteristics of the study (randomized and blind), the amount of radiation to which he or she will be exposed (even if in the control group), the scheduled clinical exams (every 3 months for 15 months), the functional tests (walking, balance, etc.), and the echo-Doppler and MRI exams at the start of the study and after 6 and 12 months.

The patient will sign the valid consent for participation in the study at the following meeting, to be agreed upon with the neurologist in charge and to be held within 15 days of the pre-randomization meeting. In any case, the valid consent must be completed and signed before MRI is performed.

b) Ascertainments from the protocol

The neurologist in charge plans the appropriate ascertainments called for by the protocol to verify that the patient's clinical conditions are compatible with inclusion in the study.
These exams are:

echo-Doppler for the diagnosis of CCSVI
laboratory tests: haemochrome, creatinine clearance, electrophoresis of proteins, glycaemia, tests to evaluate coagulation

c) Consent and clinical examination

Once the preceding procedures are completed, the neurologist in charge checks the test results (within 15 days of the pre-randomization meeting) and verifies that all of the criteria for inclusion in the study have been satisfied.

In the case that one (or more) of the inclusion criteria is not satisfied, the neurologist informs the patient that he or she is not eligible.

If the patient is eligible, the neurologist seeks to answer any questions and acquires the signed informed consent.

The neurologist performs the clinical examination and prescribes MRI.

The neurologist agrees in advance with the neuroradiology department to perform MRI within the time required by the study protocol.

In the case that the clinical exam and/or basal function tests coincide with a clinical relapse, these will be postponed at least three months to guarantee that the basal measurements are acquired when the disease is inactive. It will also be necessary to repeat verification of the inclusion criteria.

d) MRI and basal exams
MRI and the basal tests must be performed no more than 15 days before randomization.

e) Randomization
After the basal exams (see paragraph d), the neurologist contacts the center responsible for randomization to include the patient in the list. Randomization will be carried out on the same day as the intervention and will be communicated by the center responsible for randomization to the physician responsible for the angioplastic intervention in the angiography room. Both the physician responsible for the intervention and the personnel who collaborate in the procedure will be expected not to divulge the study arm to which the patient is assigned.

Randomization and the angioplastic intervention must be performed no more than 15 days after the basal exams.

The clinical pre-evaluation file, the computerized documentation and the results of the exams, the basal file and all of the clinical documents, and the sources of the data reported in the CRF must be kept by the neurologist in charge and made available to the monitor of the CRO on occasion of monitoring visits.

For the protocol for performing the basal functional tests and basal MRI, see appendix 3.

11. ECHO-DOPPLER ULTRASONOGRAPHY

See also Appendix 1

12. VENOGRAPHY AND BALLOON VENOUS ANGIOPLASTY PROCEDURE, STANDARD AND SIMULATED (SHAM)

All of the patients with RRMS/SPMS who were diagnosed with CCSVI (according to the echo-colour Doppler criteria previously described) will first be randomized to the procedure (real or simulated) and will then undergo a diagnostic venographic procedure that calls for the selective catheterization of the lumbar veins, the left renal vein, the azygous vein and the jugular veins to check for the presence and location of venous malformations defined as:

1. stenosis defined as reduction of the vasal calibre by more than 50% or a trans-stenosis gradient greater or equal to/ $\geq$ 2.2 cm of H_2O [median/mean of the trans-stenosis pressure gradient measured in patients operated on for CCSVI (88)]
2. presence of septums or obstructive or subobstructive membranes
3. inversion of valve direction
4. torsion of a venous segment with resultant stenosis
5. hypoplasia of a venous segment
6. agenesis of a venous segment
7. anomalous presence of inverted flow with respect to the physiological direction (e.g., in supine position, azygous vein that empties into the left renal vein instead of into the precava/ superior vena cava)

The single phases of the procedure are described in appendix 2.

Before the angioplastic intervention, the physician-surgeon in charge will contact the randomization center to know to which arm the patient is assigned.
The randomized patients included in the experimental arm for performance of angioplasty will receive, in the same session as the diagnostic venography, the angioplastic intervention.

Given the amount of radiology to which even the participants in the control arm will be exposed, the proponents have considered the possibility of carrying out a complete simulated procedure, up to the diagnostic phase. It was concluded that a complex performance would be needed, in which numerous professionals participate, lasting about 60-90 minutes, with the patient awake and collaborating. For this reason such a simulation would be extremely difficult, if not impossible, and would put the blindness of the study at risk without any practical advantage in the reduction of operative risk on the part of the control patient. Therefore, it was decided to subject all participants to the same treatment, with the exception of angioplasty in the control arm, and to modify the sample number and proportion in the arms considered for randomization.

Immediately after the intervention and in all successive contacts with the observing neurologist *neurologo rilevatore*, the patient will be asked to which group (real or simulated procedure) he or she thinks to have been assigned, for the purpose of discovering possible faults in the masking.The same question will be posed to the observing neurologists, excluding from any post-treatment follow-up activities the members of the operating group that performed the venographic procedure. In addition, all of the venographic procedures will be recorded by video and audio and submitted to audit. See appendix 4 for details.

13. POST-TREATMENT PATIENT EVALUATION

13.1. Endpoint and criteria for treatment efficacy

The present study calls for two primary endpoints, both measured 12 months after randomization, one clinical (improvement-stability-worsening/fluctuating in a concise functional endpoint) and the other of imaging (active lesions visualized with MRI).

The observation of a statistically significant difference in only one of the two endpoints (clinical or MRI) is sufficient to declare the study results positive. In the case of a contrast (favorable manifestations with one indicator and unfavorable with the other), the point of view of the synthetic clinical valuation will prevail, but the outcomes derived from the MRI indicator will be, in any case, widely and explicitly viewed and discussed.

The decision to utilize two primary endpoints emerged from the following considerations:

a) The action mechanisms hypothesized for venographic treatment of CCSVI do not allow exclusion of a clinical effect on the disease independent of the effect on the incidence of "active" lesions observed with MRI.
b) On the other hand, MRI parameters are largely utilized as indicators of action in clinical trials for MS and are considered "robust" / "strong" with respect to any placebo effects.
c) Given the psychometric limits of the EDSS as a measure of disability in MS (20) and the need for a long time to evaluate any changes attributable to a treatment effect, an original clinical endpoint is utilized (see 12.1.1), defined in terms of improvement/stability/ worsening on the basis of modifications observed in multiple clinical/instrumental (or functional)

measurements. This endpoint should have an adequate sensitivity to the clinical effects of treatment but has statistical properties that are not entirely known.

d) The validity of the clinical endpoint is influenced by the maintenance of blindness on the part of the patients and investigators.

The use of two primary endpoints in this situation offers at least two advantages:

a) If the complete masking / blinding of the patients and investigators during the course of the study is confirmed, the demonstration of a "significant" effect of the treatment even on only one of the two endpoints would be sufficient to refute the hypothesis that venous angioplasty is not active or efficacious in treating MS.

b) If failure of the masking/blinding procedures should be observed, which would weaken the evidence of the clinical endpoint, the evaluation of the MRI endpoint would maintain its validity entirely, thereby permitting the study to conserve one, if only partial, scientific and clinical utility.

13.1.1. Functional clinical endpoint

The clinical endpoint will be obtained through the integration of functional indicators measured with instrumental and clinically observable methods. *misurati con metodica strumentale e clinicamente rilevanti*. These are related to: walking, balance, manual dexterity, sphincteral control, and visual acuity. Each patient (at 3, 6 and 12 months after randomization) will undergo a re-evaluation using a battery of tests. On the bases of a threshold normal/pathologic value and a clinically significant difference upon follow-up (both predefined), the function will be classified as "improved/stable/worsened/fluctuating," and the integrated evaluation of the five functions will allow for the classification of the patient as improved/stable/worsened/fluctuating.

For each functional indicator the following values are defined *a priori:*

A) BASELINE
Baseline values are distinguished as normal and pathologic conditions (vision, manual ability, balance, walking) by means of cut-off definitions from the receiving operator curve (ROC**)** for the maximal correct classification or, where the cut-off just described is unavailable, for a level inferior to two standard deviations (SD) with respect to the normal average.
As regards bladder function, a cut-off is defined among pathologic conditions of different clinical conditions: indication for catheterization (cut-off for post-urination remainder > 120 ml with respect to < 120 ml).

B) VARIATION
The minimal statistically detectable difference with respect to the baseline value for $p<0.05$ (+/-2 SD) with respect to the normative data in the literature) is considered, checking for the baseline values.

The algorithm of integration among the classifiable results of the primary instrumental outcomes for the definition of "improved/stable/worsened/fluctuating" case will be as follows:

- presence of only improvement in one or more functions = improved patient
- presence of stability in all functions = stable patient
- presence of only worsening in one or more function = worsened patient
- concomitant presence of improvement and worsening in one or more functions = fluctuating patient

A more detailed evaluation of the extent of individual improvement could be conducted successively by comparing, between treatment and control groups, the improvements based on the number of improved or worsened functions.
No comparisons will be made based on the number of variations, on the population averages.

Sensitivity analysis will be performed, in which all of the lost patients will be considered, and at follow-up.

The classification of the integrated clinical endpoint will be carried out by the Clinical Endpoint Commission (CEC), which will evaluate blindly all of the documentation of each patient.The director of the Center for Training and Coordinating of Functional Measures will take part in this commission. The operative details are described in appendix 3.

13.1.2. MRI endpoint

Analysis of the MRI parameters will evaluate/reveal the "active lesions," that is, the lesions that absorb the contrast medium, the new T2 positive lesions, and the T2 positive lesions that could be of larger dimension than at the initial ascertainment. Reading of the MRI will be performed in a single center, which will carry out the reading blindly with respect to the group that executed the treatment. Performance of the MRI exams will be made by the participating centers, in keeping with the technical protocol. For classifying and operative details, see appendix 3.

13.2. Treatment safety and adverse events

The performance of a complete venographic procedure exposes the patient to ionized radiation at an efficacious dose that ranges from a minimum of 25 mSv to a maximum of 50 mSv (dose comparable to a number of chest x-rays between 1,250 and 2,500).

The doses of radiation administered to patients will be monitored during the study.

Adverse events
An adverse event is defined as a symptom, sign or undesirable clinical condition that appears after the start of the study, whether or not it is related to a concomitant treatment or surgical procedure that is part of the study.

Information relative to all of the adverse events must be recorded on the appropriate data collection form, providing the following:
• description of the event;
• duration (starting and ending dates);
• degree of severity;
• correlation with concomitant medicines under study; *con i farmaci concomitanti in studio;*
• actions undertaken (treatment, diagnostic tests); and
• outcome.

All of the adverse events must be treated until they are resolved.

Among the possible adverse events related to this study are:
1. allergic reactions or hypersensitivity to the contrast medium (immediate or delayed), such as nausea, vomiting (even prolonged), itching, hives, bronchiospasm (in 3 % of cases);

2. during dilatation of the vein: slight local pain (40% of cases), cephalea (10%);
3. during cannulation of the femural vein: minor bleeding with local haematoma at the injection site (5% of cases), nausea and lowering of arterial pressure (0.2%); and
4. post-operative cephalea.

Serious adverse events
A serious adverse event, or a serious adverse reaction, is defined as a sign or symptom or undesirable clinical condition having the following requisites:
1. it is fatal;
2. it is life-threatening;
3. it requires or prolongs hospitalization; or
4. it leads to invalidity (serious or prolonged) or to a disability.

All of the details about any serious adverse event, even if they cannot be not correlated with the intervention under study, will be reported on the Serious Adverse Event form.

Among the possible serious adverse events in this study are:
1. allergic reactions or hypersensitivity to the contrast medium (immediate or delayed), such as hypotensive shock, pulmonary edema, or cardiorespiratory arrest (1 patient in 1,000); and
2. venous thrombosis or rupturing of the vein with post-operative haemorrhage (3-4% of cases).
In cases in which a serious adverse event occurs that the experimenter judges to be related to a concomitant pharmalogical treatment, on the basis of the normative law in force regarding surveillance of pharmaceuticals *vigente in tema di farmacovigilanza* , the "Single form for reporting suspected adverse reaction" must be completed and sent to the director for drug control of the appropriate health care agency.

In addition, "unexpected" adverse events are possible, caused by technical difficulties during the venographic and angioplastic procedures.

Adverse events attributable to drugs used concomitantly for MS, for post-operative anti-tev prophylaxis and for intercurrent diseases, will be monitored to differentiate them from those associated with the experimental treatment.

13.3. Calendar of evaluations

13.3.1. Follow-up : Programmed check-ups

During the course of the study, which will last 15 months, the patient will undergo programmed check-ups on the part of the neurologist in charge at 3, 6, 9, 12 and 12+3 why not 15? months from the venographic/angioplastic intervention.

a) Duties of the neurologist in charge:

The neurologist in charge is responsible for the compilation of the CRF with:

- clinical and anamnestic updating, recording of new attacks and EDSS measurement;
- notification of adverse events to the director for drug control and to the appropriate Ethical Committee;
- recording in the CRF of adverse events and their management;
- recording of immunomodulating or immunosuppressive therapies, symptomatic /physiotherapy relationship *intercourse*, including any steroid cycles for attacks;* and

- recording of any intercurrent diseases, related ascertainments and therapies.
-

*N.B.: no immunomodulating or immunosuppressive therapy or medicines that could modify the functional measures of the clinical endpoint (symptomatic drugs) can be initiated during the course of the study (see appendix 3 for a list of these drugs).

b) Echo-Doppler monitoring:
At 6 and 12 months the patient will undergo an echo-Doppler exam to evaluate the possible presence of restenosis of the veins. The investigator will perform a blind exam and will not communicate the outcome to the patient.

c) MRI monitoring:
MRI exams will be performed at 6 and 12 months.
The participating centers will have to guarantee adherence to the MRI protocol and save every image, as called for in the protocol, to be sent for centralized image reading.

d) Monitoring of pharmacological therapies in course:
This monitoring will be carried out according to the standard criteria, which should be the same for all participating centers.

e) Final physical examination and diagnostic verification of functional tests:
Both for patients following the protocol and those excluded after randomization and maintained in the follow-up, the final physical examination will coincide with the last programmed check-up (12 + 3 months). 15 months?

13.3.2. Follow-up: Unprogrammed check-ups

Unprogrammed check-ups will be performed every time one of the following circumstances arises:
- appearance of new symptoms that suggest a new attack;
- appearance of adverse events / an adverse event?; or
- appearance of clinical events (e.g., intercurrent pathologies) that require the intervention of the neurologist in charge.

In these cases the patient should immediately contact the neurologist in charge, who will plan a physical exam as soon as possible, and in any case within 24 hours of the notification.

Unprogrammed check-ups will be carried out also in the presence of conditions that impose deferment of the functional tests (see appendix 3).

a) Duties of the neurologist in charge:
- The same as reported in point 12.3.1.a.
- Prescribes steroid treatment for relapses or other treatments for intercurrent pathologies.

b) Attack follow-up:
Upon the judgment of the neurologist in charge, successive examinations will be programmed for the purpose of defining the further evolution of an attack, in particular the period of stabilization of symptoms and the score of the residual EDSS.

14. RULES FOR UTILIZING AND PUBLICIZING DATA

The study was constructed according to the criteria of independent clinical research defined by the Italian Pharmacologic Agency (**AIFA)** for studies of pharmaceuticals. The Steering Committee is responsible for collecting and analyzing the data. The Promoter of the study, the University Hospital Administration of Ferrara (in keeping with the DM of 17 December 2004), owns the data and assumes responsibility for the successive publication of the study results.

A copy of the protocol will be submitted to the internationally recognized trial registries (clinicaltrial.gov, WHO register, etc.).

No presentation or diffusion of the preliminary results is foreseen at the conclusion of the study. Any preliminary communications to congresses or conferences will have to be approved by the Steering Committee and will regard the study design only.

Divulgence of any news related to the study, by whatever means of communication, as well as communication at cultural or congressional events, will take place only through the Scientific Director or the Chairman of the Steering Committee, provided that they agree, and with the consent of the majority of the members of the Steering Committee.

Media release of the results will be exclusively via the press office of the Azienda Ospedaliera Universitaria di Ferrara.

Appendix 1. Protocol for ultrasonologic investigation/examination

Neurosonologic diagnosis of CCSVI

A. Echo-Colour Doppler of the internal jugular veins (IJVs) and vertebral veins (VVs)

The exam begins with the patient in a resting position, under comfortable climatic conditions and with low lighting. At first the patient is in a position of clinostatism (back-rest at 0°), then sitting (back-rest at 90°) (for the easiest evaluation of anatomic relationships, congenital and acquired alterations, and the tributary veins of the jugular group in this latter position). If a tilt-chair is not available, it is advised to rest the patient's head and trunk on a rigid surface. At least two minutes must pass between the two testing conditions, and the patient must have performed several deep respirations.
The internal jugular and vertebral venous system will be examined.
Each center will have to indicate the productive characteristics and technical specifications of the echograph used, and also guarantee that the parameters are within the requested investigative fields/areas (for each single study, in addition to iconographic documentation, the values of the parameters indicated below will be reported to the investigator).
The instrumentation must allow for real-time digital acquisition of the exam.

Technical characteristics of the echo-Doppler

- Linear trasducer 5-11 MHz
- B-mode exam

Frequency 7-11 MHz for studying the jugular district and 5-9 MHz for studying the vertebral venous plexus
Study in fundamental harmonic and/or second harmonic (indicate on each exam which of the two and with what frequency for both)
Steering of the ultrasonic bundle/beam (trapezoid – 56°)
TGC and gain adjusted singularly as needed (indicate for each exam)

- Exam in Colour-mode or in Power-mode with directional modality

Frequency 3-5 MHz
PRF 1.5-2.5 kHz
Automatic steering of box (trapezoid)
PRF adjusted singularly on the basis of flow velocity in the spectrogram (indicate on each exam the velocity scale used as marker)
Correction of the angle will not be made in relation to variability in course or evaluation in longitudinal scanning of the venous structures and, in any case, evaluations will be made on the flow spectrum analysis**.**

1. **linear** 3.5-10 MHz used for scanning the veins of the neck (internal jugular veins and veins of the vertebral plexus)

The ultrasonic exam is carried out for both patients and controls with the subject in the supine position, with the head in line with the neck and in slight hyperextension (the head may be only slightly rotated in the opposite direction from the side to be examined), and then with the subject in a sitting position. *controdirezionalmente*

B-mode evaluation

This evaluation has two purposes:

1. to mark *reperire* sectional areas that are particularly narrow, to the extent of no longer being measurable (see measurement of sectional area); and
2. to mark the presence of intraluminal anomalies that cause flow disturbances if not total blockage (see reflux evaluation).

The study is conducted in transversal and longitudinal scanning, starting with the valvular plane and proceeding for the entire extracranial course. The transducer is positioned in the region above the clavicle, at the juncture between the IJV and the brachiocephalic vein, identifying the valvular structures as a reference point.
A preliminary evaluation of the jugular valvular apparatus at rest is carried out in B-mode, and of the echographic aspect of the valves, sometimes eccentric.

The malformations of the IJV are:
a. presence of septums;
b. anomalous valves; and
c. double canal (of which one is without blood flow).

N.B.: Attention should also be paid to the presence of the vagus nerve, which runs posteriorly to the jugular vein in the proximal section examined, and to the presence of the thoracic duct. At times these two structures, if not identified, can lead to an erroneous diagnosis of an anatomic venous anomaly.

Measurement of the area of the jugular veins

A first evaluation of the patency of the IJV is performed in transversal scanning.
In this scanning, applying a minimal pressure on the skin with the transducer in order not to alter the calibre of the vein itself and with direction exactly perpendicular to the transducer, the cross-sectional area of the IJV is measured (manual tracking on frozen image).
The marker/reference point? is the common carotid artery (CCA) at the level immediately preceding the bulbar, which must appear circular, not elliptical, in cross-section, to determine thereby the perpendicularity of the transducer. The IJV appears / is located along the side of the carotid artery and under the sternocleidomastoideus; following it in direction of the cranium, one finds the external carotid on the side.
The IJV is examined in correspondence to 3 points of reference:
J1: distal jugular (valvular plane);
J2: median jugular (at the level of the scutum: larynx process); and
J3: proximal jugular (jugular point, in correspondence with the carotid bifurcatio).

The measurement of the cross-sectional area (CSA) is carried out (at the end of the expiratory phase) at points J1, J2 and J3, with the patient breathing spontaneously.
The measurement of the area at the J2 level is used to evaluate/calculate the difference in supine-sitting area (criterion 5: negative difference).

Evaluation of reflux

The IJVs and VVs are examined for possible reflux.
The **internal jugular veins** are at first evaluated in transversal and longitudinal scanning. Flow direction is evaluated by means of the colour parameter and by positioning the sample volume inside

the vessel, comparing it to the flow direction in the carotid. Subsequently, the flow direction is evaluated in longitudinal scan.

During normal respiration, any inversion in flow direction is recorded at the end of the inspiratory phase (criterion 1: presence of reflux constantly present for at least 1 second). The presence of any flow blockage is evaluated (criterion 4). Spectrographic documentation must be indicated of such a finding, in consideration of the fact that on the spectrum it is possible to calculate the temporal parameter with precision.

The **vertebral veins** (VV) are evaluated in longitudinal scanning. After the vein (or veins) and the vertebral artery (in V2, in correspondence to the common carotid artery, with visualization of the vertebral artery in the intertransversalis process anatomical relationship) have been visualized, flow direction is evaluated and any reflux (criterion 1) or flow blockage (criterion 4) is recorded.
Reflux (criterion 1) is considered to be even/also the finding of blood flow directed from the vertebral vein and/or the extrarachidian plexuses towards the intrarachidian plexuses.
(even in this case, the two modalities, Colour and/or Power-mode and Doppler must both be used and documented).

B. Echo-colour Doppler of the intracranial veins (ICV)

Technical characteristics of the echo-Doppler

Echograph with presetting for transcranial color Doppler sonography **TCCS** spell out? and modifications for studying the nervous system.
- Sectorial/sectional transducer, 2-2.5 MHz
B-Mode
Sectorial/sectional scanning with sector from 90°
Depth of exam of at least 15 cm (visualization of contralateral theca cranica?)
Anterior-posterior orientation defined by the identification of the epiphysis on the diencephalic plane (on the right half of the scanning)
Axial scanning with oblique planes for the venous structures
Frequency 1.5-2.5 MHz
TGC in grey scale and gain adjusted singularly on the basis of visualization
Imaging in fundamental harmonic
Medium/Average wall filter
Colour-mode or Power-mode in directional modality
Frequency 1.5-2.0 MHz
PRF 0.375-0.7 kHz
Gain adjusted singularly
Doppler (duplex or triplex mode)
Frequency 1.5-2.0 MHz
Gain adjusted singularly
PRF adjusted on the basis of flow velocity in the spectrum (indicate scale used for each exam)
No angle correction for the parameters of the spectrogram

Evaluation of the presence of reflux in the intracranial/intracerebral veins (ICV)

Following a preliminary evaluation of the arteries of the cranialis base, useful for determining the quality of the acoustic window, the PRF is reduced to the lowest acceptable value, and the gain in colour or power is modified to attain the best signal/noise ratio. The depth is such as to visualize the contralateral theca cranica on the side to be examined.

Rosenthal's basal vein is found in the postpeduncular tract, situated near the posterior cerebral artery (tracts P2 and P3), with flow moving away from *con flusso in allontanamento* the transducer (for both the posterior cerebral artery and Rosenthal's vein).
Flow inversion in Rosenthal's vein is evaluated during spontaneous respiration, in any moment of the respiratory cycle (criterion 2: flow inversion in the ICVs; also in this case, Doppler documentation with flow spectrum or multigate system becomes necessary).
The flow spectrum is also evaluated, reporting the flow direction and velocity (**PSV** and **EDV**), at the level of the transverse sinus, bilaterally, paying attention to the frequent conditions of hypoplasia of the same. Flow normally moves in the direction away from the transducer. The diagnosis of flow inversion is evaluated, also in this case, in any phase of the respiratory cycle.
It is also possible to evaluate the contralateral cavernous sinus through the condyle window, and to analyze flow direction with the multigate system.

Appendix 2. Protocol for venography, angioplasty and the simulated procedure

Venography and angioplasty

Preparation

- Preparation of angiography room with an automatico injector preloaded with iodized non-ionic contrast medium at a concentration of 300 mgI/ml
- Cannulation of a peripheral vein of the arm
- Positioning of patient supine on radiology bed
- Subcutaneous local anaesthesia at the level of the left inguinal fold with 10 ml of kainic acid? *xilocaina* at 2% concentration?
- Preparation of a sterile working field

Diagnostic procedure

- Positioning of a valved introducer *introduttore valvolato* 7-9 Fr, 15 cm long, using Seldinger's technique, in the left femoral vein
- Administration of 5,000 units of sodic heparin intravenously
- The venographic study proceeds in PA projection of the left femoroiliac venous ***asse*** and of the inferior vena cava with an angiographic catheter 4-5 Fr (20ml at a flow of 4 ml/s), aimed at identifying any stenosis from the compression of the left common iliac vein or of the vena cava, with consequent collateral circles through the left ilio-lumbar and left ascending lumbar veins.
- Thus, by means of ascending catheterization of the left ilio-lumbar vein, spinal subtractive digitalized venography of the lumbar tract is performed in PA projection, in order to examine the anatomy of the intrarachidian and extrarachidian venous plexus and its flow. For this reason, injection of the contrast medium is made with flows (4ml/s) and quantities (20-30 ml) capable of opacifying that venous bed without provoking an artificial diversion of flow. In case of difficult catheterization of the left ilio-lumbar vein, a lateral sacral vein may be catheterized, or a lumbar vein directly. The principal purpose of this study is point out large/gross/serious *grossolane* congenital or acquired alterations, in the form of atresias, ageneses, or stenoses of the extrarachidian venous drainage.
- The successive study calls for retrograde catheterization and subtractive digital venography of the left renal vein (flow 4 ml/s; quantity 15 ml) capable of evidencing any stenosis at the

outlet of the vena cava, with diversion of the renal venous return in the rachidian system through the reno–azygous–lumbar connection.

- The procedure continues by bringing the angiographic catheter into? the superior vena cava and measuring the venous pressure by means of a manometer.
- Next, the outlet of the azygous vein into the superior vena cava is catheterized for a few millimeters, and retrograde mdc/contrast medium/agent? is injected manually in order to visualize the possibility of refluent opacification of the vein and to document any valves or septums stenosing the lumen.
- With the aid of a hydrophilic guide, the catheter is moved inside the azygous vein until it is proximal to the confluence with the hemiazygous vein, and venous pressure is measured.
- Subtractive digital azygousgraph is performed in Right Posterior Oblique projection (15°-25°) with appropriate flow (3 ml/s) and quantity (10 ml) of mdc/contrast medium/agent to respect the normal anterograde flow of the vein and to identify any refluxes or stenoses in correspondence with the arch of the azygous vein.
- The most complete morphological study, retrograde, of the azygous vein can be carried out with greater flow (8 ml/s) and quantity (30 ml); in this way, it is possible to opacify entirely the system of origin of the azygous vein and the hemiazygous, up to the ascending lumbar vein.
- The procedure continues with venographic study of the internal jugular veins. A manual injection of mdc/contrast medium/agent is made to evaluate the continence of the jugular valves; catheterization of the jugular is performed using a hydrophilic guide, requiring the patient to perform deep inspirations, and possibly the Valsalva manoeuvre, in order to facilitate venous emptying and opening of the valves. After the catheter has been moved in the jugular vein to the level of the mandibular angulus/angle/corner, local venous pressure is measured, followed by retrograde subtractive digitalized venographic study (flow 3ml/s, quantity 8 ml) of the internal jugular veins in PA projection, possibly completed in Oblique or Orthogonal projection. Also in this case, the purpose of venography is to detect alterations in the jugular outlet, in the form of septums, annular or conoid stenoses, atresias or ab-extrinsic compressions, with evidence of venous stagnation. The field examined must also include the corresponding brachiocepahalic venous trunk and the superior vena cava, also potentially affected by stenosing alterations.
- Finally, retrograde catheterization is carried out on the vertebral veins, confluent in proximity to the jugular vein, venography of which is performed manually.

Interventive procedure on the azygous vein

- The presence of anatomic anomalies at the level of the arch of the azygous vein, such as stenoses, endoluminal membranes or septums, and torsion (twisting), that necessitate an endovascular correction are dilatated by means of angioplastic catheters of a compliant type for increased flexibility, which allows for their insertion in the narrow arch of the azygous vein. Balloons 8-10 mm in diameter and 2-4-6 cm long are utilized, insufflated to a maximal pressure of 8 atmospheres (atm). These insufflations are maintained for 30-60 seconds and are repeated several times.
- At the end of the therapeutic procedure the angiographic catheter is reinserted, and the manometric measurement and azygousgraph are repeated, in order to document the morphological outcome of the treatment. *atte a documentare*
- If retrograde venography demonstrates stenoses even more caudad to the arch of the azygous vein, and thus in the thoracic tract of the azygous and hemiazygous veins, these also can be dilatated through the angioplastic procedure, but with balloons of a smaller calibre (5-6 mm in diameter), possibly utilizing small-calibre catheters (3 Fr) and appropriate guides (.018 inch).

Interventive procedure on the jugular veins

- Correction of such jugular alterations is performed by means of angioplastic procedure, which is normally begun through the use of compliant-type balloons 10-12 mm in diameter and 20-40 mm in length, insufflated to 8 atm; smaller-diameter balloons are used in case of significant/serious hypoplasias or atresias. In the first instance, the compliant-type balloons are selected not only for dilatative treatment, but also for determining the consistency of the stenosis, evidenced by the appearance of an impression or fossa on the profile of the balloon at its maximal dilatation.
- If the post-procedural result is unsatisfactory, dilatation is completed through use of noncompliant-type balloons at high pressure (18-20 atm) and 10 mm in diameter. These insufflations are maintained for 30-60 seconds and repeated several times.
- The angioplastic catheters are introduced with the help of an angiographic guide ***da cambio*** (260 cm in length; .035 inch in diameter?), possibly of the stiff type in cases with greater tortuosity. Any stenoses of the brachiocephalic venous trunk must also be corrected via angioplasty, with balloons of even greater calibre (14 mm).
- At the end of the therapeutic procedure, the angiographic catheter is reinserted and venous manometric measurement and venography are repeated in order to document the morphological outcome of the treatment.
- When the catheter in the brachiocephalic venous trunk is withdrawn, **oppure**
While the catheter in the brachiocephalic venous trunk is being withdrawn,
another manual injection of the contrast medium is performed to evaluate whether the competence/patency? of the valvular borders/limbuses has been maintained or not after angioplasty.

Interventive procedure on the vertebral veins

- In the event that retrograde catheterization of the vertebral veins shows stenoses, which are rather rare, angioplasty is carried out with balloons 8-10 mm in diameter and 20-40 mm in length, inflated/insufflated to 8 atm.
- At the end of the procedure, the valved introducer is removed, and manual haemostasis is performed at the site of the femoral venous puncture, on which a compressive bandage is applied, and the patient remains in bedrest for a few hours.
- The appearance of any complications will be monitored after 24 hours and again after one week.
- The patient will receive therapy with low molecular weight heparin according to the dosage prescribed by the operator.

SIMULATED ANGIOPLASTIC PROCEDURE

INTERVENTIVE PROCEDURE ON THE AZYGOUS VEIN or JUGULAR VEINS

- The operators in the angiographic room will simulate all of the actions described in the real procedure, explaining to the patient what they are doing in each phase and telling the patient what he/she might feel, but reassuring him/her that it is normal not to feel anything.
- The real and simulated procedures do not differ except in the performance of angioplasty.
- It is essential to guarantee absolute blindness, doing whatever necessary to separate the operators from the patients (in both groups/arms of the study) as much as possible, paying attention to ensure patient safety. There should be no contact of any type between the

operators and the patients at the end of the procedure, except for the management of any adverse events.

The appearance of any complications will be monitored after 24 hours and again after one week.

- Instead of low molecular weight heparin, a placebo will be administered. Both the active drug and the placebo will be provided in appropriate quantity to the patients, and these must be prepared so that they are indistinguishable. During check-ups it must be ensured that the patient has not used different drugs that could seem to be the same. **not clear-**

that resemble either the heparin or the placebo?

Appendix 3. Protocol for functional clinical evaluation and MRI

Functional clinical evaluation

Functional indicators are defined as measurements related to the degree of interaction between the person as a whole and the environment. These are not measurements, therefore, of intracorporeal functions such as the conduction velocity of nerve impulses or the concentration of plasmatic electrolytes.

The maximal degree of "objectivity" is attainable, in theory, if an inanimate object measures another inanimate object. The minimal degree of objectivity occurs when an animate subject judges another animate subject. In the case of functional measures of a person, maximal objectivity is excluded. However, there are "intermediate" situations in which: a) the measurement is, in any case, performed by an inanimate instrument; and b) the judgment is made by the person himself or herself. *dalla persona stessa*.

In these cases the uncertainty of the estimation/bias? is lower than that foreseen/which is likely in the "external judgment" of a person.

Choice of the indicator for the proposed study

To maximize the objectivity of the measurements (according to what was stated above), they are differentiated as follows:

- variables in the primary endpoint, measurable with physical instruments; and
- variables in the secondary endpoint, measurable with other- or self-administered questionnaires.

In choosing the indicators, the following factors were taken into account:

a. the prevalence and clinical importance of the variables;

b. the objectivity (with the limitations mentioned above) of the measurements;

c. the compatibility of the statistical properties of the measurements with a reasonable sample size; and

d. the overall feasibility of the tests in terms of tolerability, time, costs and necessary competence.

An extensive study has been conducted of the literature, taking into account the specific experience acquired in the field of functional measurements, by various components of the Working Group. The decisions then resulted from joint discussions and reflections.

The following proposal was reached:

Measurements by physical instruments (Indicators of primary endpoint)

Variables in Outcome-Indicators of primary endpoint	Test	Modality-Indicator
**Balance**	Balance Master-Limits of stability (LOS) In statics on dynamometric platform, oscillate to reach positions imposed by the pressure center	MXE-Maximal End-point Excursion[1]

**Walking**	Walking on floor at a spontaneous speed. For 10 m, chronometric measurement of time; counting of number of steps<br>Calculation of walk ratio: relationship of step length to frequency	Numerical indicator (ratio nondimensional) [2]
**Manual dexterity**	Manual dexterity. Box & Block test. One-handed movements of wooden cubes.	Number of blocks trasferred in one minute[3]
**Sphincter control**	Post-urination remainder, echographic (e.g., Bladder Scan).	Volume (in ml) post-urination remainder[4]
**Visual acuity**	Visual acuity - Low Contrast, Sloan Letter Chart (Precision Vision Inc.- contrast 100%, 2.5%, 1.25%).	Numbers of lines not legible at various contrast levels [5]

1. Jbabdi et al. BMC Geriatrics 2008, 8:8. Available from: http://www.biomedcentral.com/1471-2318/8/8
2. Tesio et al. Multiple Sclerosis (in press)
3. Paltamaa et al. Arch Phys Med Rehabil 2007;88:1649-1657
4. Kelly. Reviews in Urology 2004;6 (SUPPL. 1):S32-S37
5. Balcer et al. Neurology 2007;68:1299–1304

Measurements via questionnaires (Secondary endpoint: * Self-administrated; ^ Other-administrated)

Variables in Outcome-Indicators of secondary endpoint	Test	Modality-Indicator
**Emotive state (anxiety/depression)**	*Questionnaire HADS A and D, cumulative points	score, range 0-84 [6]
**Cognitive level**	^Questionnaire MoCA mental state	score, range 0-30 [7]
**Bladder hyperactivity (urgency, pollakiuria, urge incontinence)**	*Overactive Bladder Questionnaire-b. Bladder hyperactive/urgency - "bother" subscale	score, range 8-48 [8]
**Fatigue**	*Questionnaire M-FIS (Modified-Fatigue Impact Scale)	score, range 0-84 [9]
**Macro-staging of disability in** Multiple Sclerosis	^EDSS-Extended Disability Status Scale<br><br>^PASAT – Paced Auditory Serial Addition Test	score, range 0-10 (2-5.5 in inclusion criteria)[10]<br><br>absolute score (number of exact answers) Range 0-240[11]

6. Honarmand K, Feinstein A. Validation of the Hospital Anxiety and Depression Scale for use with multiple sclerosis patients. Mult Scler 2009;15,12:1518-1524.
7. Nasreddine ZS, Phillips NA, Bedirian V, Charbonneau S, Whitehead V, Collin I, et al. The Montreal Cognitive Assessment, MoCA: a brief screening tool for mild cognitive impairment. J Am Geriatr Soc 2005;53,4:695-699.
8. Coyne KS, Matza LS, Thompson CL, Kopp ZS, Khullar V. Determining the importance of change in the overactive bladder questionnaire. J Urol ,2006;176,2:627-632.
9. Kos D, Nagels G, D'Hooghe MB, Duportail M, Kerckhofs E. A rapid screening tool for fatigue impact in multiple sclerosis. BMMC Neurol 2006;6:27.
10. Hobart J, Freeman J, Thompson A. Kurtzke scales revisited: the application of psychometric methods to clinical intuition. Brain 2000;123 (Pt 5):1027-1040.
11. RA Rudick , Polman CH, Cohen JA et al. Assessing disability progression with the Multiple Sclerosis Functional Composite. Multiple Sclerosis 2009; 15: 984–997.

The following have been defined for all of these instruments:
- bibliographical sources;
- normality ranges;
- expected variance in the population under examination;
- sensitivity to change (minimal variation measurable with significance $p<0.05$ and/or minimal clinically important variation);
- test-retest stability (in particular, nonspecific "practice effect" for motor test repetition);
- accessibility and costs of the instrumentation on the market (to measure balance, bladder remainder, and visual acuity);
- psychometric validation and availability of valid Italian version (for the questionnaires);
- commitments of time and energy required for patients and operators; and
- commitment required for training of operators.

Design of the measuring procedures

"Practice effect" and "stable base line"
The measurements of balance, walking *locomozione,* and manual dexterity are susceptible to inducing improvement due to subjects' learning the procedures in repeated trials (**c.d.** "practice effect"). This phenomenon risks generating improvements not brought about by therapy and not generalizable to activities different from the test itself. Nevertheless, there are valid reasons for asserting that, for the proposed indicators, a significant practice effect is lacking/the practice effect is insignificant provided that at least two or three weeks pass between test repetitions. Considering that the study calls for randomization of cases treated with angioplasty vs. a ke" procedure, it would be possible to avoid the procedure (common in quasi-experimental study designs for behaviour variables) of verifying the stability of the functional measures in at least two observations prior to treatment. Whenever a minimal practice effect might be present, it would in any case be divided between the two treatment groups.

Definition of variations

As specified elsewhere in the protocol, the design aims at determining the incidence of "improved, stable, worsened and fluctuating" cases, and not average quantitative variations in single parameters.
For each functional indicator, the following values are defined *a priori:*

A) BASELINE:
baseline values discriminating between pathological and normal conditions (vision, manual ability, balance, walking) through definition of a cut-off value on the ROC curve to maximize correct classification, or, where the cut-off just described is unavailable, a lower limit of -2 SD **??** with respect to the normal average. *per livello inferiore a 2SD rispetto a media normativa*.
As for bladder function, a cut-off value has been established to distinguish pathological conditions of different clinical significance from an indication for catheterization (cut-off for post-urination remainder >200 ml). This cut-off takes into account a possible and documented echographic overestimate of 20 ml at the most, and exceeds the 100 ml limit established in all of the neuro-urological literature.

Instrumental indicators that contribute to the primary endpoint "improved/stable/worsened patient"			
Indicator	Cut-off for normality and minimal observable difference	Pre-selected quantitative criteria: cut-off for normality; statistically significant variation, where possible, also clinically	Main bibliography (another available)
Visual acuity.<br>Reading of 8 types, at low contrast, on lines with progressively smaller characters	Normality: reading of lines at high contrast corresponding to 20/20 acuity. 2 lines less at 2.5% contrast; 2 lines less at 1.25% contrast<br>Minimal statistically detectable variation: 2 lines	Normality: reading of all proposed lines at high contrast (20/20). Differences not defined at low contrast (indicatively 2 lines less at 2.5%, another 2 less at 1.25%)<br><br>Statistically detectable: variation of 2 lines.<br>Clinically, 2 lines predictive compared to Natalizumab's efficacy (reduced worsening compared to nontreated, and predictivity compared to variations in QoL scores)	Mowry EM,et al. Vision related quality of life in multiple sclerosis: correlation with new measures of low and high contrast letter acuity. J Neurol Neurosurg Psychiatry. 2009 Jul;80(7):767-772.
Vesical-sphincter function: emptying ability<br>Echographic measurement of post-urination remainder	Normality: remainder < 20 ml.<br>Minimal detectable variation: 20 ml	Clinically important cut-off: 120 ml remainder; lower than this not specific for intermittent catheterization<br><br>Clinically significant variation:<br>– basal remainder >201 ml:50 ml<br>– basal remainder < 200: 30 ml	Kalsi V, Fowler CJ. Therapy Insight: bladder dysfunction associated with multiple sclerosis. Nat Clin Pract Urol. 2005;2(10):492-501.
Manual dexterity (Box & Block Test).<br>One-handed moving of wooden cubes, in the predetermined time of one minute	Normalit': 60 (SD 12).<br>Intrasubject, recognizable variation of 9 cubes	Normality cut-off: 36<br><br>Statistically recognizable: 9 cubes<br>Clinically: reduction of 9 cubes corresponds to EDSS progression of 1 point	Goodkin DE et al.Comparing the ability of various compositive outcomes to discriminate treatment effects in MS dinical trials.Multiple Sclerosis 1998;4: 480-486.
Balance. In statica, ability to oscillate in different directions. Balance Master Test, "maximal polydirectional excursion" parameter - "composite" score for 4 orthogonal directions (c.d. MXE comp)	Normality: 97/100 (SD 6)<br>Recognizable variation of 12	Normality cut-off: 85<br>Statistically recognizable: 12 points<br>Clinically: variation of 12 already obtained with rehabilitative treatment	Williams NP, Roland PS, Yellin W. Vestibular evaluation in patients with early multiple sclerosis. Am J Otol 1997;18:93-100.
Walking. step length/frequency ratio ("walk ratio-WR")	Data on healthy subjects: 6.48±0.68<br><br>Data on MS population: 5.19 ±0.83	Normality cut-off:<br>lower 95° percentile, healthy: 5.66<br><br>Significant variation: 2 SD healthy=0.68*2=1.36	Sekiya N, Nagasaki H. Reproducibility of the walking patterns of normal young adults: test-retest reliability of the walk ratio(step-length/step-rate). **Gait Posture**. 1998; **7,3:225-22.??**<br>Specifically for MS: Tesio L et al. J Rehabil Med 2010, submitted.

B) VARIATION:
minimal statistically detectable difference compared to baseline value for $p<0.05$ (+/-2 SD compared to the normative data in the literature), checking for baseline values.

The algorithm of integration among the classification outcomes of the instrumental primary endpoint that will allow for classification of each individual case as "improved/stable/worsened/ fluctuating" will be established as follows:

- improvement in one or more functions, the others being stable = improved patient
- stability in all functions = stable patient
- worsening in one or more function, the others being stable = worsened patient
- concomitant presence of improved and worsened functions = fluctuating patient

Post-hoc/ After this, a more in-depth comparison of the individual improvement entity/factor could be conducted by comparing, among treated cases and controls, classes of variation based on the numbers of improved/worsened functions.

No comparisons will be made based on the number of variations calculated on population averages.

Criteria for potential postponement or inadmissibility of functional evaluations

Functional measurements are potentially sensitive to clinical events or interventions that influence the disabilities that affect/underlie?? behavioural performance. The use of ortheses or aids is considered below, in a separate paragraph.

A. *Insurgence of disabilities and/or their treatment*

Clinical-therapeutic events can occur that alter the functional abilities of the MS patient or that can be an indirect, not demonstrable manifestation of it. the disease? For the purposes of this study, each situation is regulated as shown below.

A.1 Events that exclude one or more functions from every successive evaluation

**Event type**	**Test to postpone or exclude**
Insurgence of major neurological and/or cognitive disabilities of another nature than MS (stroke, polyneuritis, labyrinthitis, etc.)	**All**
Insurgence of orthopedic disability that permanently alters the mobility of the lower limbs (e.g., fracture)	Balance<br>Walking
Insurgence of orthopedic disability that permanently alters the mobility of the upper limbs (e.g., fracture)	Manual dexterity
Insurgence of disability that permanently alters vesical-sphincteric function (e.g., prostatectomy)	Urinary retention
Insurgence of disability that permanently alters visual function (e.g., cataract, detached retina)	Visual acuity at low contrast<br>Balance<br>Manual dexterity

Insurgence of psychiatric symptoms (e.g., need for antipsychotic therapy)	Functional tests on questionnaires

A.2 Events that call for evaluation postponement

For some conditions, postponement of the evaluations, rather than exclusion, can be foreseen. These include minor orthopedic disabilities (e.g., tibiotarsal distorsion, wrist fracture, post-traumatic hydrarthrosis), all transitory, and/or other minor intercurrent pathologies, such as vesical and opthalmological problems (cystitis, conjunctivitis, etc.). As regards the correspondence between the type of disability and the functions that cannot be evaluated, reference should be made to the Table in point A.1.

A.3 Definitive renouncement or postponement of the evaluation

A.3.1 For the conditions listed in point A.1, the operators should abandon definitively the evaluations specified in the Table.

A.3.2 For the conditions listed in point A2, the operators should postpone the evaluations for at least 90 days after the insurgence of the disability and also ensure that, at the time established for the evaluation, at least 30 have passed since the resolution of the symptoms.
purché al momento previsto siano trascorsi 30 giorni dalla risoluzione dei sintomi.

A.3.3 If in two evaluative sessions (even nonconsecutive) , one or more functional measurements must be renounced, or one or more functional measurements must be postponed, the patient will no longer be able to undergo functional measurements and will be considered worsened in those functions that could not be measured, even if only once.
An example could be a patient who first suffers a tibiotarsical distorsion and then cystitis, and who has to postpone the corresponding evaluations on two successive occasions. In this case, the patient would be considered worsened for the purposes of walking, balance, and sphincter control.
He or she would proceed only with the evaluations of manual dexterity and visual acuity, as well as the findings of the secondary endpoint in the questionnaires.
nonché con le rilevazioni di endpoint secondari su questionari.

A.4 Relapses
The patient must be free of relapses for at least three months. If the programmed check-up coincides with a clinical relapse, the patient will have to be re-evaluated after three months.

A.5 Beginning of, or variation in, rehabilitative treatments (motorial-cognitive therapeutic exercises and/or physical therapies)

The patient must not modify his/her regime of rehabilitative treatment. This term signifies a set of neuromotorial treatments of a certain type, intensity and frequency, with motorial or cognitive exercises, and/or physical therapies, which are typically performed by each patient on an out-patient basis at rehabilitative centers, or else in home-assistance regimes.
Following is a glossary of nine different typologies into which the above treatments can be grouped:

1. Individual motorial therapeutic exercise (manual and/or instrumental) with direct contact with supervisor

2. Group motorial therapeutic exercise (even/also instrumental) with supervision

3. Individual or group cognitive therapeutic exercise

4. Sphincteral therapeutic exercise (including biofeedback) and/or tibial electrostimulation (**PTNS**) **spell out?**

5. Motorial electrostimulation

6. Analgesic electrotherapy, acupuncture, analgesic laser therapy, massotherapy, thermic or diathermic therapy

7. Iono- or iontophoresis

8. Manual medicine, massotherapy, mechanotherapy (including vibration; continuous passive mobilization, etc.)

9. Other therapy within the area of complementary medicine, for analgesic or rehabilitative purposes (specify) ________________________________

Psychotherapeutic interventions and/or personal or family counselling will not be considered aims of this study.

<u>It is agreed that "treatment regime that must not be modified" includes all of the following</u>:

a) average duration of the therapeutic sessions (on a trimester basis), whatever the therapeutic typologies: *quali che siano le tipologie di terapia che vi rientrano* the allowed variation is a maximum of 30 minutes/session (on the average) **not clear, if max is 30, the average must be lower than 30**
b) frequency of the sessions (cumulative calculation on a trimester basis): 20% allowed variation
c) setting of the sessions: only out-patient or home-assistance or rehabilitative Day Hospital are admissible, or combinations thereof. Rehabilitative hospital stay is not admissible (see below).
d) treatment "mix": any combination of the typologies listed above
numerical variation: maximum 2 typologies
typological variation even without numerical variation: maximum 2 typologies

Any patient who modifies his/her treatment regime must leave the study.

A.6 Hospital admission
Hospital admission to <u>acute care departments</u> *unità per acuti* (e.g., Neurology) are permitted if there are none of the functional outcomes or after-effects listed in points A1 and A2; however, hospital admissions for rehabilitation are not permitted.
Any patient who is admitted to a hospital for rehabilitation (not in Day Hospital) must leave the study.

A.7 Occasional rehabilitative treatments
It is permissible for study patients to undergo a sporadic cycle of treatment, be it motorial or using physical means, for transitory motorial impairments not correlated with MS, provided that the treatment ends at least one month prior to the evaluation. An example would be treatment for arthrotic joint pain.

A.8 Use of ortheses and aids (including lenses)
Motorial ortheses and aids (e.g., orthotics for the foot, walking cane) must be removed if not necessary to perform the functional tests. If the patient has an absolute need for a cane or other aid, the walking and/or balance tests will not be admitted. If the neurologist considers them necessary, other ortheses (excluding walking aids) will have to be maintained during all of the successive functional evaluations, even if the patient no longer finds them indispensable.

If, after the start of the study, the patient finds it absolutely necessary to use a motorial orthesis or aid to perform a functional evaluation, or else an orthesis or aid different from that used initially due to the insurgence or worsening of a persistent disability, the patient will be considered worsened in that function even if the orthesis or aid utilized renders the function possible. Variation in the use of lenses between one evaluation and a successive one will be permitted (if in keeping with what is specified in point A.1). The type of correction and its variations must be recorded in the CRF.

A.9 Symptomatic medicines
Some medicines can have an influence specifically on the above-mentioned impairments. Following is a list (probably incomplete) of these medicines:

1. intrathecal antispasmodics
2. botulinic toxin
3. oral antispasmodics
4. anti-tremor medicines?? *antitremorigeni*
5. psychotropic medicines in general
6. steroids
7. pharmacological modulators of vesical- sphincteral control (anticholinergics, alphalytics).

The beginning of intrathecal antispasmodic treatment (case 1) after recruitment results in a "worsened" classification in the integrated instrumental functional endpoint.

After recruitment, the start of botulinic toxin therapy (case 2), if applied to a lower limb, renders the walking and balance tests inadmissible and, ifapplied to an upper limb, renders the manual dexterity test inadmissible. For the corresponding functions, the evaluations will be considered impracticable.

All other pharmaco-therapies cited are admissible, provided that they are: a) present at the start of the study and not modified during its course; or b) begun during the course of the study but terminated at least one month prior to the functional evaluation. In all other cases, the discovery of use of these therapies is reason to postpone the motorial (cases 3,4,5,6, EDSS, MSFC) or sphincteral (case 7) evaluations.

8. Neurostimulatory implants
Implantation of a sacral neuromodulator or an epidural stimulator after recruitment results in a "worsened" classification in the integrated instrumental functional endpoint.

9. Operators' tasks
The operators must carry out all possible measurements, citing all current and previous therapies in the three months preceding the first measurement and in the interval between each measurement and the preceding one.

10. Pathogenetic medicines

It should be recalled that the criteria for general exclusion from the study include therapy with natalizumab or fingolimod, and current or previous therapy with cladribine and/or laquinimod. The initiation, once the study has begun, of immunomodulating and/or immunosuppressive therapies of any type must be avoided and, in any case, recorded on the CRF.

Therapies that do not interfere with MS disease activity nor with the cited impairments are admissible, e.g., antibiotics and antihypertensives.

Reports on functional indicators and operating manual

Measurements will be performed blindly, as regards treatment, by two resident investigators at the participating center. These investigators will receive specific training at the Center for Training and Coordinating of Functional Measures.
Detailed descriptions of the tests and measurement and interpretation procedures will be included in a Study Manual to be provided to the experimenters. This will contain:
a) a concise description of the test protocols?? *prove dei test* and the rationale for choosing them;
b) a reference bibliography; and
c) test performance instructions. *un manuale di esecuzione dei test.*
Aside from the functional measurements called for in the protocol, the investigators must have no diagnostic-therapeutic relationships with the patients for the duration of the study.

In addition, throughout the study, the functional data operators must not relate either test results or their impressions to anyone. In particular, they must avoid asking information of any type about the study patients from personnel involved in the angiographic procedure.
The test results must be inserted in the CRF immediately after they are attained; they cannot be inserted periodically in groups.

MRI Evaluation

MRI PROTOCOL

Introduction

Magnetic resonance imaging (MRI) will be performed throughout the study at the participating centers with the same equipment and with magnets operating at a minimum of 1.5 Tesla. Any updating of the MRI hardware and software must be reported to the MRI analysis center.

In accordance with previous verification of hepatorenal function, MRI will be carried out with or without the contrast means.

The MRI protocol must call for a locator to establish the position of the patient, who is checked using a rapid sequence. Finally, two series are carried out? for the quantitative analysis of the lesions, one DP/T2 sequence and one T1 sequence, weighed/weighted after administration of the contrast medium/agent. **e se mdc non viene usato?**

Dummy scan

Each center must perform a "dummy scan" comprised of two separate image acquisitions before beginning the study. This dummy scan will be used to evaluate some parameters regarding the quality of the images, that is, the accuracy of the repositioning and the signal/noise ratio of the images.

The dummy scan will be performed using the parameters of the standard protocol described in the following section, with these exceptions:

1) the contrast medium will not be administered; and
2) the T1 series will be acquired with the same parameters as the post-contrast T1 series (even though the contrast means will not be administered to the control group).

After the first "dummy run," the patient will be made to get off of the bed, and after about 30 minutes, the entire sequence of repositioning and image acquisition will be repeated.

Patient preparation

1. Evaluate scrupulously any contraindication to performance of the exam (for example, metallic implants, pacemakers, defibrillatory implants, infusion pumps for medicines).
   This phase is of enormous importance to patient safety and is part of the procedures routinely applied in MRI centers. The study protocol must not interfere with these aspects of patient safety.
2. Explain the procedure to the patient.
3. Locate a venous access.

4. Provide adequate hearing protection (headphones or plugs for the ears)
5. Position the patient in comfortably on the bed.
6. Stabilize the neck and head.
7. Use the laser to line up the coil. *bobina* The horizontal ray must be parallel to the orbital protuberance, and the vertical ray must pass through the point of the nose.

MRI equipment

General notes

1. Parallel imaging: acquisition in parallel imaging (SMASH, SENSE, ASSETT and similar) are not permitted in this protocol.
2. Use of multi-channel coils: in this case, use "intensity correction" if available.
3. When several TE values are possible, utilize the lowest one available.
4. Do not use interpolation options.

Positioning technique

1) After having acquired a rapid locator on three levels, us the internal auditory canal *canale acustico interno* as an anatomic point of reference for the correct positioning of right-left rotation and the interhemicerebral fissure for a correct coronale positioning; at this point, acquire a scout on the sagittal plane.
2) Use the acquired sagittal image to prescribe the axial slices that will have to cover the entire encephalon/brain/cerebrum, from the apex to the foramen magnum. The prescription will have to be angled in such a way that the central slice touches the lower border of the genu and of the splenium of the corpus callosum.

Sequence for evaluating the brain:

1) Weighted T1 volumetric images
2) At the end of this acquisition, the contrast medium/agent (gadolinium-DTPA 0.1 mmol/Kg) will be administered intravenously

Weighted images in DP/T2:

- dual-echo fast/turbo spin echo (FSE/TSE)
- NEX: 1
- echo train length: 8-16
- TR 2200-3000 ms
- TE/TE2: 30-50 ms, 60-100 ms
- Number of slices necessary to obtain coverage of the foramen magnum at the apex
- Slice thickness: 4 mm
- Slice gap: 0; interleaved
- FOV 250 (or FOV rectangular reduced by a factor of 75% in the direction of phase encoding)
- Matrix: 256 x 256
- Direction of phase encoding: L-R

3) **Weighted T1 images** (obtained after administration of gadolinium-based contrast medium/agent)

- conventional spin echo sequences
- NEX: 2
- TR 600-650 ms
- TE: 10-20 ms
- Number of slices necessary to obtain coverage of the foramen magnum at the apex
- Slice thickness: 4 mm
- Slice gap: 0; interleaved
- FOV 250 (or FOV rectangular reduced by a factor of 75% in the direction of phase encoding)
- Matrix: 256 x 256
- Direction of phase encoding: L-R
- Flow compensation: on

Data archiving

Each center must conserve a copy of all of the digital images. In addition, a copy of all of the digital data must be sent to the Center for Centralized MRI Reading and to the Data Coordination Center. Printed copies and x-ray plates *lastre* should not be sent.

MRI reading

MRI will be performed blindly. The following parameters will be evaluated for every timepoint:

- New hyperintensive lesions in the weighted T2 sequences.
- New lesions that present contrast absorption in the T1 images after gadolinium administration.
- Total number of lesions that present contrast absorption in the T1 images after gadolinium administration.
- Number of lesions that present "enlarging" in the weighted T2 sequences.
- Total volume of the hyperintensive lesions in the weighted T2 sequences.
- Total volume of the hypointensive lesions in the weighted T1 sequences.
- Cerebral volume cerebrale ("cerebral atrophy")
- Lesions that are contemporaneously new T2 and "enlarging" and gadolinium-enhancing will be counted only once.

Two expert neuroradiologists will evaluate the images and identify and count the lesions in mutual agreement. A single lesion that absorbs contrast is defined as a contrast-enhanced area seen on a certain weighted T1 axial slice, and is not referable either to structures that normally absorb contrast nor to contrast inside vascular structures.

On the T2 and DP-weighted FSE/TSE images, a single hyperintensive lesion is defined as an area of increased signal on a certain axial slice and is not referable to structures that are normally hyperintensive. New T2 and DP-weighted hyperintensive lesions must appear in areas in which no abnormal signal was present in the preceding exam.

A lesion is defined as "enlarging" when a single hyperintensive lesion in the T2 and DP-weighted sequences on a certain slice shows increased dimensions with respect to the preceding exam.

A single hypointensive lesion in T1 is defined as an area characterized by an intermediate signal intensity between those of the grey matter and the cerebrospinal fluid.

New hypointensive T1 lesions must appear in the areas in which a surely identifiable hypointensity was not present in the preceding exam.

Contiguous lesions in adjacent axial slices will be counted only once.

Lesional volumed and calculations of the cerebral volume will be conducted by expert personnel who will use specifically designed software.

Quality control

The quality of the MRI data will be evaluated by the Center for Centralized MRI Reading. Within three days of arrival of the data to the Center, the images will be evaluated, and any need to repeat an exam or part of it will be communicated to the appropriate MS center.

Appendix 4. Accreditation criteria for participating MS centers and quality control

a) Accreditation criteria for participating MS centers

The Center for Vascular Diseases of the University of Ferrara Hospital will act as Coordination and Promotion Center.* Proponent and Coordinating Center. To this Center will be referred all training for the participating centers that relates to neurosonologic diagnosis of CCSVI and venographic and angioplastic procedures. **è così all'inizio del documento*

Training for clinical endpoint measurements will be referred to the Center for Training and Coordinating of Functional Measures.

Center for Experimental Design and Definition of Functional Measures?? **not mentioned above**

Each center participating in the study will be an MS center in itself and competent in at least four areas: neurology, neuroradiology, noninvasive vascular diagnosis, invasive/interventive vascular radiology, and psychometric or rehabilitative functional therapy. ?

Every center involved must be accredited by the National Healthcare Service for the procedures included in the study, even **if/though** the procedures are funded entirely by the study project.

Participation by the MS centers and the related **UU.OO.?** angiographic and sonologic centers will be approved by an ad hoc commission (comprised of the Scientific Director, the President of the Steering Committee, a neurologist, a sonologist, and a radiologist).

The MS center must have at least 400 patients under its care and must contribute a minimum of 30 and a maximum of 50 subjects to the study.

The director of the MS center will have to document that the center has participated, in the past five years, in at least one multi-centered randomized controlled trial and has acquired sufficient experience with randomized experimental designs and blindness on the part of the patients and the clinician in charge.

For each of the areas of competence listed above, the characteristics that permit accreditation of the center must be reported on the multi-centric map of the study.

Competence in neurology. The neurologist in charge of the study at each center must:

- present documentation that attests his/her participation, in the past five years, in at least one multi-centric randomized controlled trial and, if not the director of the MS center, must attach authorization from the center's director;

- present a CV complete with his/her publications related to MS and in indexed journals. *e su riviste indicizzate*. He/she is required to have at least one publication in which he/she appears as coauthor of one randomized controlled trial;

- commit to guaranteeing the recruitment of the number of patients agreed upon with the **PI** of the project; and

- guarantee that he/she will follow the study protocol scrupulously.

Competence in noninvasive vascular diagnosis. This competence is given to professionals who can collaborate with the MS center and thus are in the same hospital district or its immediate vicinity. In Italy this competence does not belong to a clearly defined specialist since vascular diagnosis with ultrasound is performed by vascular surgeons, angiologists, cardiologists, radiologists, and by neurologists known as neurosonologists. Each of these specialists who habitually carries out evaluations of vascular ultrasonography and who makes himself/herself available to the study can be part of the center's team. To these two qualifications, the specialist will have to add a basic course specific to vascular diagnosis of CCSVI, to be offered exclusively at the Center for Vascular Diseases of the University of Ferrara. After the course there will be a period of self-teaching with at least 50

normal and 30 pathological exams, plus a recall to be carried out in Ferrara. Finally, the investigator will have to pass an examination which consists of applying the protocol blindly at the above-mentioned MS center.

Competence in interventive radiology and/or endovascular surgery. Venous angioplasty can be performed by one or both of these specialists. They must be part of the same hospital district where the MS center is located or in its immediate vicinity. They must present continual, certified activity in interventive vascular radiology. The physician-radiologist who will carry out venography and the angioplastic intervention will have to be a professional who works autonomously and who has performed, since the start of his/her training curriculum and as first operator, at least 150 procedures overall in the invasive diagnostic and invasive therapeutic-interventive sectors, with an case-mix percentage of not less than 20%, indicatively, for therapeutic-interventive activity. Maintenance of clinical competence on the part of the specialist must call fore the performance of at least 240 procedures overall in the two above-mentioned sectors, with a case-mix percentage of not less than 20%, indicatively, for therapeutic-interventive activity, every three years. (Standard called for by the Specific Requisites in Radiology for accreditation in the Emilia Romagna region). For specific training in the performance of the venographic and venous angioplastic procedures, the professional will have to participate in the conducting of at least five procedures flanked by Dr. Galeotti of the Interventive Radiology module of the University of Ferrara Hospital.

Competence in the psychometric-functional area. The instrumental and psychometric functional measurements will be conducted by personnel belonging to one or more of the following professional specialties:
- physicians
- physiotherapists
- occupational therapists
- speech therapists
- psychologists
- psychiatric rehabilitation technicians
- nurses

The center will give preference to operators who already have experience in the field of neuromotorial and vesical-sphincteral measurements and, subordinately, measurements in the psychological-cognitive area. The operators will receive specific training for the measurements called for in the protocol.

b) Training of operators in the functional-rehabilitative area. Center for Training and Coordinating of Functional Measures

A Center for Training and Coordinating of Functional Measures will be established/identified, having a director for the activities connected with the present study.
This Center will need to have the following requisites:

- an Operating Unit in Physical and Rehabilitative Medicine (or other equivalent denomination);
- medical research activity at an institutional level;
- competence in: physiatrics, physiotherapy, speech therapy, neuropsychology, nursing, and neurology;
- research laboratories active in the neuromotorial, cognitive and psychometric areas;
- direct experience in functional tests of the type called for in the study; *sperimentazione* and
- documented activity in research and scientific publication related to functional and instrumental measurements of disability.

The director of the Training Center will need to have personal experience in clinical and laboratory management regarding disability and rehabilitation, as well as in functional evaluative methodology. The director will coordinate the activities of the Clinical *Functional* Endpoint Committee. *cf pg. 1*

The tests necessary for the measurement of the concise functional endpoint and the secondary endpoints (aside from the EDSS, done by the neurologist) will be conducted by operators from *afferenti* the recruitment centers/participating MS centers?, indicatively two per center, who will be specifically trained by the Center for Training and Coordinating of Functional Measures.
This latter Center will provide training and accreditation for functional indicator operators, who will be asked to participate in an ad hoc one-day course for theoretical and theoretical-practical instruction.

Learning will be verified by means of a theoretical-practical test in which a __**?**_____ will be distributed concerning the histological procedures and the procedures for and assignment of measurement. When the operators have tested about 30% of the study subjects, they will return to the Training Center for a refresher course-test to evaluate how well they have retained the competence acquired.
Each recruitment center will be provided with the necessary equipment.

The Center will also make telephone and e-mail assistance available to the operators in the field in case of necessity.
In consideration of the time-frame of the study, the two operators will by included in an annual contract, at a distance of six months from one another. Therefore, there will be six months during which both operators will be assigned to the same center and during which it will be possible to provide vacation time to each, as well as to guarantee adequate data delivery. **(?)**

c) Collection of clinical information
All of the information reported in the CRF of the study will have to be recorded in the usual clinical documentation of the patient. This documentation should include when participation in the study was proposed to the patient. It will be the duty of the CRO monitor to verify the congruence of all information.
As regards the functional data, the operators will have to enter their data on-line immediately after the measurements. The Training Center will provide the CRO and the Data Coordination Center with the criteria for evaluation of completeness and internal/entire?? coherence of the measurements. The CRO will proceed to contact the operators concerning any incomplete data and will transmit any incoherent data to the Training Center, which will contact the operators.

The Training Center will organize at least one "site visit" within the time of recruitment of the 15th case for each center in order to verify in the field the correctness of protocol application.
The walking, balance, and manual dexterity tests will be video-recorded, according to standards defined by the Training Center, and sent to the CRO, which will make them available to the Training Center for any verification request, on any patient sample requested by that Center, which will, in any case, view at least 5% of the videos. The Training Center will remain, however, blind with respect to the treatment received by the patients.

d) Verification of masking/blindness during the course of the study
Given the study design and the clinical outcomes considered, maintenance of masking/blindness is crucial to the evaluation of treatment efficacy.
At every check-up during the follow-up period, the neurologist in charge, the operator responsible for measuring the clinical endpoint, and the patient will be asked which treatment (real or false/sham) they think has been given. This investigation will offer information regarding the maintenance of the masking/blindness.
For the purpose of detecting possible criticisms *criticità* of the masking/blindness, all procedures will be video- and audio-recorded.

15. Bibliography

1) Abo-Krysha N, Rashed L. The role of iron dysregulation in the pathogenesis of multiple sclerosis: an Egyptian study. *Mult Scler* 2008; 14(5):602-608.

2) Adams CW, Abdulla YH, Torres EM, Poston RN. Periventricular lesions in multiple sclerosis: their perivenous origin and relationship to granular ependymitis. Neuropathol Appl Neurobiol 1987; 13(2):141-152.

3) Adams CW. Perivascular iron deposition and other vascular damage in multiple sclerosis. J Neurol Neurosurg Psychiatry 1988; 51(2):260-265.

4) Adhya S, Johnson G, Herbert J, Jaggi H, Babb JS, Grossman RI et al. Pattern of hemodynamic impairment in multiple sclerosis: dynamic susceptibility contrast perfusion MR imaging at 3.0 T. Neuroimage 2006; 33(4):1029-1035.

5) Al Omari MH, Rousan LA. Internal jugular vein morphology and hemodynamics in patients with multiple sclerosis. Int Angiol 2010; 29(2):115-120.

6) Bartolomei I, Salvi F, Galeotti R, Salviato E, Alcanterini M, Menegatti E et al. Hemodynamic patterns of chronic cerebrospinal venous insufficiency in multiple sclerosis. Correlation with symptoms at onset and clinical course. Int Angiol 2010; 29(2):183-188.

7) Betts CD, D'Mellow MT, Fowler CJ. Urinary symptoms and the neurological features of bladder dysfunction in multiple sclerosis. Journal of Neurology Neurosurgery and Psychiatry 1993;56(3):245-250.

8) Boldrini P et al. Minimal record of disability and environmental situation in MS in the province of Ferrara. Rivista di Neurologia 1987; 57:65-69.

9) Brass SD, Chen NK, Mulkern RV, Bakshi R. Magnetic resonance imaging of iron deposition in neurological disorders. Top Magn Reson Imaging 2006; 17(1):31-40.

10) Cadavid D, Tang Y, O'Neill G. Responsiveness of the Expanded Disability Status Scale (EDSS) to disease progression and therapeutic intervention in progressive forms of multiple sclerosis. Rev Neurol 2010 Sep 16;51(6):321-329.

11) Carmosino MJ, Brousseau KM, Arciniegas DB, Corboy JR. Initial evaluations for multiple sclerosis in a university multiple sclerosis center: outcomes and role of magnetic resonance imaging in referral. Arch Neurol 2005; 62(4):585-590.

12) Compston A, Coles A. Multiple sclerosis. Lancet 2008 Oct 25; 372(9648):1502-1517.

13) Confavreux Ch, Vukusic S. Age at disability milestones in multiple sclerosis. Brain 2006; 129:595–605.

14) Cottrell DA, Kremenchutzky M, Rice GP, Hader W, Baskerville J, Ebers GC. The natural history of multiple sclerosis: a geographically based study. 6. Applications to planning and interpretation of clinical therapeutic trials in primary progressive multiple sclerosis. Brain 1999 Apr; 122(Pt 4):641-647.

15) Cottrell DA, Kremenchutzky M, Rice GP, Koopman WJ, Hader W, Baskerville J, Ebers GC. The natural history of multiple sclerosis: a geographically based study. 5. The clinical features and natural history of primary progressive multiple sclerosis. Brain 1999 Apr; 122(Pt 4):625-639.

16) Craelius W, Migdal MW, Luessenhop CP, Sugar A, Mihalakis I. Iron deposits surrounding multiple sclerosis plaques. Arch Pathol Lab Med 1982; 106(8):397-399.

17) De Keyser J, Steen C, Mostert JP, Koch MW. Hypoperfusion of the cerebral white matter in multiple sclerosis: possible mechanisms and pathophysiological significance. J Cereb Blood Flow Metab 2008; 28(10):1645-1651.

18) De Stefano N, Matthews PM, Filippi M, Agosta F, De Luca M, Bartolozzi ML et al. Evidence of early cortical atrophy in MS: relevance to white matter changes and disability. Neurology 2003; 60(7):1157-1162.

19) Doepp F, Friedemann P, Valdueza MJ, Schmierer K, Schreiber SJ. No cerebro-cervical venous congestion in patients with multiple sclerosis. Annals of Neurology 2010. Epub. http://dx.doi.org/10.1002/ana.22085.

20) Ebers GC, Heigenhauser L, Daumer M, Lederer C, Noseworthy JH. Disability as an outcome in MS clinical trials. Neurology 2008; 71:624-631.

21) Ebers GC, Koopman WJ, Hader W, Sadovnick AD, Kremenchutzky M, Mandalfino P, Wingerchuk DM, Baskerville J, Rice GP. The natural history of multiple sclerosis: a geographically based study. 8. Familial multiple sclerosis. Brain 2000 Mar; 123 Pt 3:641-649.

22) Fleiss JL. Analysis of data from multi clinic trials. Controlled Clinical Trials 1986; 7:267–275.

23) Frischer JM, Bramow S, Dal-Bianco A, Lucchinetti CF, Rauschka H, Schmidbauer M, Laursen H, Sorensen PS, Lassmann H. The relation between inflammation and neurodegeneration in multiple sclerosis brains. Brain 2009 May; 132(Pt 5):1175-1189. Epub 2009 Mar 31.

24) Ge Y, Jensen JH, Lu H, Helpern JA, Miles L, Inglese M et al. Quantitative assessment of iron accumulation in the deep gray matter of multiple sclerosis by magnetic field correlation imaging. AJNR Am J Neuroradiol 2007; 28(9):1639-1644.

25) Ge Y, Zohrabian VM, Osa EO, Xu J, Jaggi H, Herbert J et al. Diminished visibility of cerebral venous vasculature in multiple sclerosis by susceptibility-weighted imaging at 3.0 Tesla. J Magn Reson Imaging 2009; 29(5):1190-1194.

26) Goldberg JD, Koury KJ. Design and analysis of multicenter trials. In: Berry DA, ed. Statistical methodology in the pharmaceutical sciences. New York: Marcel Dekker, 1990, pp. 201–37.

27) Haacke EM, Cheng NY, House MJ, Liu Q, Neelavalli J, Ogg RJ et al. Imaging iron stores in the brain using magnetic resonance imaging. Magn Reson Imaging 2005; 23(1):1-25.

28) Haacke EM, Garbern J, Miao Y, Habib C, Liu M. Iron stores and cerebral veins in MS studied by susceptibility weighted imaging. Int Angiol 2010; 29(2):149-157.

29) Haacke EM, Makki M, Ge Y, Maheshwari M, Sehgal V, Hu J et al. Characterizing iron deposition in multiple sclerosis lesions using susceptibility weighted imaging. J Magn Reson Imaging 2009; 29(3):537-544.

30) Henderson APD, Barnett MH, Parratt JDE, Prineas JW. Multiple sclerosis: distribution of inflammatory cells in newly forming lesions. Ann Neurol 2009; 66:739–753.

31) Hirtz D, Thurman DJ, Gwinn-Hardy K et al. How common are the "common" neurologic disorders? Neurology 2007; 68(5): 326-337.

32) Hojnacki D, Zamboni P, Lopez-Soriano A, Galleotti R, Menegatti E, Weinstock-Guttman B et al. Use of neck magnetic resonance venography, Doppler sonography and selective venography for diagnosis of chronic cerebrospinal venous insufficiency: a pilot study in multiple sclerosis patients and healthy controls. Int Angiol 2010; 29(2):127-139.

33) Inglese M, Adhya S, Johnson G, Babb JS, Miles L, Jaggi H et al. Perfusion magnetic resonance imaging correlates of neuropsychological impairment in multiple sclerosis. J Cereb Blood Flow Metab 2008; 28(1):164-171.

34) Khan O, Filippi M, Freedman MS, Barkhof F, Dore-Duffy P, Lassmann H et al. Chronic cerebrospinal venous insufficiency and multiple sclerosis. Ann Neurol 2010; 67(3):286-290.

35) Kozinska D, Holland CM, Krissian K, Westin CF, Guttmann CR. A method for the analysis of the geometrical relationship between white matter pathology and the vascular architecture of the brain. Neuroimage 2004; 22(4):1671-1678.

36) Kremenchutzky M, Cottrell D, Rice G, Hader W, Baskerville J, Koopman W, Ebers GC. The natural history of multiple sclerosis: a geographically based study. 7. Progressive-relapsing and relapsing-progressive multiple sclerosis: a re-evaluation. Brain. 1999 Oct;122 (Pt 10):1941-50.

37) Kremenchutzky M, Rice GP, Baskerville J, Wingerchuk DM, Ebers GC. The natural history of multiple sclerosis: a geographically based study 9: observations on the progressive phase of the disease. Brain. 2006 Mar;129(Pt 3):584-94. Epub 2006 Jan 9.

38) Krogias C, Schröder A, Wiendl H, Hohlfeld R, Gold R. "Chronic cerebrospinal venous insufficiency" and multiple sclerosis: critical analysis and first observation in an unselected cohort of MS patients. Nervenarzt. 2010 Jun;81(6):740-6.

39) Kurtzke JF, Beebe GW, Norman JE. Epidemiology of multiple sclerosis in U.S. veterans: I. Race, sex, and geographic distribution. Neurology 1979; 29(9 Pt 1):1228-35

40) La Mantia L, Munari LM, Lovati R. Glatiramer acetate for multiple sclerosis. Cochrane Database of Systematic Reviews 2010, Issue 5. Art. No.: CD004678. DOI: 10.1002/14651858.CD004678.pub2

41) Lassmann H, Bruck W, Lucchinetti C. Heterogeneity of multiple sclerosis pathogenesis: implications for diagnosis and therapy. Trends Mol Med 2001;7: 115–21.

42) Lee AB, Laredo J, Neville R. Embryological background of truncular venous malformation in the extracranial venous pathways as the cause of chronic cerebro-spinal venous insufficiency. Int Angiol 2010; 29(2):95-108.

43) LeVine SM, Lynch SG, Ou CN, Wulser MJ, Tam E, Boo N. Ferritin, transferrin and iron concentrations in the cerebrospinal fluid of multiple sclerosis patients. Brain Res 1999; 821(2):511-515.

44) LeVine SM. Iron deposits in multiple sclerosis and Alzheimer's disease brains. Brain Res 1997; 760(1-2):298-303.

45) Lublin FD, Reingold SC Defining the clinical course of multiple sclerosis: results of an international survey. National Multiple Sclerosis Society (USA). Advisory Committee on Clinical Trials of New Agents in Multiple Sclerosis. Neurology 1996; 46(4):907-911

46) Lucchinetti C, Bruck W, Parisi J, Scheithauer B, Rodriguez M, Lassmann H. Heterogeneity of multiple sclerosis lesions: implications for the pathogenesis of demyelination. Ann Neurol 2000;47:707-17

47) Marrie RA, Rudick R, Horwitz R, Cutter G, Tyry T, Campagnolo D et al. Vascular comorbidity is associated with more rapid disability progression in multiple sclerosis. Neurology 2010; 74(13):1041-1047.

48) Menegatti E, Genova V, Tessari M, Malagoni AM, Bartolomei I, Zuolo M et al. The reproducibility of colour Doppler in chronic cerebrospinal venous insufficiency associated with multiple sclerosis. Int Angiol 2010; 29(2):121-126.

49) Menegatti E, Zamboni P. Doppler haemodynamics of cerebral venous return. Curr Neurovasc Res 2008; 5(4):260-265.

50) Pittock SJ, Mayr WT, McClelland et al. Disability profile of MS did not change over 10 years in a population based color.**??** Neurology, 2004; 62: 601-606

51) Plasmati R, Pastorelli F, Fini N, Salvi F, Galeotti R, Zamboni P. Chronic cerebro-spinal venous insufficiency: report of transcranial magnetic stimulation follow-up study in a patient with multiple sclerosis. Int Angiol 2010; 29(2):189-192.

52) Pugliatti M, Rosati G, Cartone H et. al. The epidemiology of multiple sclerosis in Europe. European Journal of Neurology 2006; 13:700-22.

53) Qiu J. Venous abnormalities and multiple sclerosis: another breakthrough claim? Lancet Neurol 2010; 9(5):464-465.

54) Rice GPA, Incorvaia B, Munari LM, Ebers G, Polman C, D'Amico R, Parmelli E, Filippini G. Interferon in relapsing-remitting multiple sclerosis. Cochrane Database of Systematic Reviews 2001, Issue 4. Art. No.: CD002002. DOI: 10.1002/14651858.CD002002.

55) Rosales CJ. [The significance of enlarged retinal veins in the diagnosis of multiple sclerosis: report of a patient (author's transl)]. Med Clin (Barc) 1981; 77(1):30-32.

56) Sadovnick D, Ebers GC. Epidemiology of multiple sclerosis: a critical overview. Can J Neurol Sci 1999; 20:17-29.

57) Scalfari A, Neuhaus A, Degenhardt A, Rice GP, Muraro PA, Daumer M, Ebers GC. The natural history of multiple sclerosis, a geographically based study 10: relapses and long-term disability. Brain. 2010 Jun 9.

58) Schelling F. Damaging venous reflux into the skull or spine: relevance to multiple sclerosis. Med Hypotheses 1986; 21(2):141-148.

59) Senn S. Statistical issues in drug development. New York: Wiley, 1997

60) Simka M, Kostecki J, Zaniewski M, Majewski E, Hartel M. Extracranial Doppler sonographic criteria of chronic cerebrospinal venous insufficiency in the patients with multiple sclerosis. Int Angiol 2010; 29(2):109-114.

61) Simka M, Zaniewski M. Reinterpreting the magnetic resonance signs of hemodynamic impairment in the brains of multiple sclerosis patients from the perspective of a recent discovery of outflow block in the extracranial veins. J Neurosci Res 2010; 88(9):1841-1845.

62) Simka M. Blood brain barrier compromise with endothelial inflammation may lead to autoimmune loss of myelin during multiple sclerosis. Curr Neurovasc Res 2009; 6(2):132-139.

63) Singh AV, Zamboni P. Anomalous venous blood flow and iron deposition in multiple sclerosis. J Cereb Blood Flow Metab 2009; 29(12):1867-1878.

64) Sormani MP, Bonzano L, Roccatagliata L, Cutter GR, Mancardi GL, Bruzzi P. Magnetic resonance imaging as a potential surrogate for relapses in multiple sclerosis: a meta-analytic approach. Ann Neurol. 2009 Mar;65(3):268-75.

65) Stankiewicz J, Panter SS, Neema M, Arora A, Batt CE, Bakshi R. Iron in chronic brain disorders: imaging and neurotherapeutic implications. Neurotherapeutics 2007; 4(3):371-386.

66) Swingler RJ, Compston DA. The morbidity of multiple sclerosis. Quarterly Journal of Medicine 1992;83:325-37.

67) Talbert DG. Raised venous pressure as a factor in multiple sclerosis - Medical Hypotheses. 2008;70:1112–7 (Imperial College School of Medicine, London)

68) Tan IL, van Schijndel RA, Pouwels PJ, van Walderveen MA, Reichenbach JR, Manoliu RA et al. MR venography of multiple sclerosis. AJNR Am J Neuroradiol 2000; 21(6):1039-1042.

69) Thompson EJ, Zeman A. Fluids of the brain and the pathogenesis of MS. Neurochem Res 1992; 17(9):901-905.

70) Trapp BD, Peterson J, Ransohof RM, Rudick R, Mork S, Bo L. Axonal transection in the lesions of multiple sclerosis. N Engl J Med 1998; 338: 278–85.

71) Trojano M, Pellegrini F. Fuiani A. et al. New natural history of interferon-beta-treated relapsing multiple sclerosis. Ann. Neurol. 61, 2007: 300-306

72) Tsutsui S, Stys PK. Degeneration Versus Autoimmunity in Multiple Sclerosis (editorial). Annals of Neurology 2009;66:711-13.

73) Varga AW, Johnson G, Babb JS, Herbert J, Grossman RI, Inglese M. White matter hemodynamic abnormalities precede sub-cortical gray matter changes in multiple sclerosis. J Neurol Sci 2009; 282(1-2):28-33.

74) Villoslada P, Arrondo G, Sepulcre J, Alegre M, Artieda J. Memantine induces reversible neurologic impairment in patients with MS. Neurology 2009; 72(19):1630-1633.

75) Vos CM, Geurts JJ, Montagne L, van Haastert ES, Bo L, van d, V **???**et al. Blood-brain barrier alterations in both focal and diffuse abnormalities on postmortem MRI in multiple sclerosis. Neurobiol Dis 2005; 20(3):953-960.

76) Vosoughi R, Freedman M. Therapy of MS. Clinical Nerurology and Neurosurgery, 112,2010: 365-385

77) Walter U, Wagner S, Horowski S, Benecke R, Zettl UK. Transcranial brain sonography findings predict disease progression in multiple sclerosis. Neurology 2009; 73(13):1010-1017.

78) Weinshenker BG, Bass B, Rice GP, Noseworthy J, Carriere W, Baskerville J, Ebers GC. The natural history of multiple sclerosis: a geographically based study. 2. Predictive value of the early clinical course. Brain. 1989 Dec;112 (Pt 6):1419-28.

79) Weinshenker BG, Bass B, Rice GP, Noseworthy J, Carriere W, Baskerville J, Ebers GC. The natural history of multiple sclerosis: a geographically based study. I. Clinical course and disability. Brain. 1989 Feb;112 (Pt 1):133-46.

80) Weinshenker BG, Rice GP, Noseworthy JH, Carriere W, Baskerville J, Ebers GC. The natural history of multiple sclerosis: a geographically based study. 4. Applications to planning and interpretation of clinical therapeutic trials. Brain. 1991 Apr;114 (Pt 2):1057-67.

81) Weinshenker BG, Rice GP, Noseworthy JH, Carriere W, Baskerville J, Ebers GC. The natural history of multiple sclerosis: a geographically based study. 3. Multivariate analysis of predictive factors and models of outcome. Brain. 1991 Apr;114 (Pt 2):1045-56.

82) Wink B, Baurmann H. Vascular changes in multiple sclerosis. Klin Monbl Augenheilkd 1972; 161(3):272-277.

83) Westlund KB, Kurland LT. Studies on multiple sclerosis in Winnipeg, Manitoba, and New Orleans, Lousiana. Am J Hyg 1953 May;57(3):380-96.

84) Wuerfel J, Paul F, Zipp F. Cerebral blood perfusion changes in multiple sclerosis. J Neurol Sci 2007; 259(1-2):16-20.

85) Yulin Ge, Vahe M. Zohrabian, and Robert I. Grossman. 7T MRI: New vision of microvascular abnormalities in multiple sclerosis. Arch Neurol. 2008;65(6): 812–6.

86) Zamboni P. The big idea: iron-dependent inflammation in venous disease and proposed parallels in multiple sclerosis. J R Soc Med. 2006 Nov;99(11):589-93. Review.

87) Zamboni P, Consorti G, Galeotti R, Gianesini S, Menegatti E, Tacconi G et al. Venous collateral circulation of the extracranial cerebrospinal outflow routes. Curr Neurovasc Res 2009; 6(3):204-212.

88) Zamboni P, Galeotti R, Menegatti E, Malagoni AM, Gianesini S, Bartolomei I et al. A prospective open-label study of endovascular treatment of chronic cerebrospinal venous insufficiency. J Vasc Surg 2009; 50(6):1348-1358.

89) Zamboni P, Galeotti R, Menegatti E, Malagoni AM, Tacconi G, Dall'Ara S et al. Chronic cerebrospinal venous insufficiency in patients with multiple sclerosis. J Neurol Neurosurg Psychiatry 2009; 80(4):392-399.

90) Zamboni P, Lanzara S, Mascoli F, Caggiati A, Liboni A. Inflammation in venous disease. Int Angiol 2008; 27(5):361-369.

91) Zamboni P, Menegatti E, Bartolomei I, Galeotti R, Malagoni AM, Tacconi G et al. Intracranial venous haemodynamics in multiple sclerosis. Curr Neurovasc Res 2007; 4(4):252-258.

92) Zamboni P, Menegatti E, Galeotti R, Malagoni AM, Tacconi G, Dall'Ara S et al. The value of cerebral Doppler venous haemodynamics in the assessment of multiple sclerosis. J Neurol Sci 2009; 282(1-2):21-27.

93) Zamboni P, Menegatti E, Weinstock-Guttman B, Schirda C, Cox JL, Malagoni AM et al. The severity of chronic cerebrospinal venous insufficiency in patients with multiple sclerosis is related to altered cerebrospinal fluid dynamics. Funct Neurol 2009; 24(3):133-138.

94) Zamboni P, Menegatti E, Weinstock-Guttman B, Schirda C, Cox JL, Malagoni AM et al. CSF dynamics and brain volume in multiple sclerosis are associated with extracranial venous flow anomalies: a pilot study. Int Angiol 2010; 29(2):140-148.

95) Zivadinov R, Marr K, Ramanathan M, Zamboni P, Benedict RRHB, Cutter G et al. Combined Transcranial and Extracranial Venous Doppler Evaluation (CTEVD Study). Description of the design and interim results of an epidemiological study of the prevalence of chronic cerebrospinal venous insufficiency in MS and related diseases. [P06.144] AAN Annual Meeting 2010. Toronto, 15 Apr 2010.

96) Zivadinov R, Schirda C, Dwyer MG, Haacke ME, Weinstock-Guttman B, Menegatti E et al. Chronic cerebrospinal venous insufficiency and iron deposition on susceptibility-weighted imaging in patients with multiple sclerosis: a pilot case-control study. Int Angiol 2010; 29(2):158-175.

TRANSLATION OF THE ETHICAL COMMITTEE APPROVAL OF A SUBSTANTIAL AMENDMENT FOR THE MULTICENTER RANDOMIZED STUDY FOR EVALUATION OF SAFETY AND EFFICIENCY OF A PROCEDURE OF BALLOON ANGIOPLASTY OF THE EXTRACRANIAL VEINS FOR THE TREATMENT OF MULTIPLE SCLEROSIS (PI Prof. P. Zamboni)

The substantial amendment relative to the above protocol approved on April 2011 determines the following changes:

- To reduce the sample size from 685 patients to 300 patients;
- To cancel the follow-up visit at 9 months;
- To modify the eligible study population: will be only eligible patients diagnosed with relapsing remitting multiple sclerosis.
- To update the inclusion criteria as follows:
    1) the disease duration will be extended up to 15-years for patients with relapsing remitting Multiple Sclerosis;
    2) the enrollment of patients will be possible also for subjects coming from centers linked with the center responsible for patient follow-up.
- To update the exclusion criteria as follows:
    1) elimination of natalizumab (Tysabri);
- To add in Appendix 2 the section "False Positive Management": Positive Doppler and Negative Catheter Venography paragraph;
- To update the contact information for centralized readers in order to send images for MRI assessment.

With regard to the form called "Information Sheet for the patient" it is required to:

- Delete the sentence: "Your data will be recorded even if you will not be able to participate into the trial because of inclusion and exclusion criteria."
- Add the sentence " You have to undertake, under your personal resposability, any measure to avoid the occurrence of a pregnancy for the entire study period, because cannot be excluded the possibility of malformations of the unborn.
- Add the explanation: you may discuss with the research physician the modality in order to avoid pregnancy.
- Explain the owners and persons responsible for personal data.

With regard to the form called "Consent Form" it is required to:

- Enter the sentence "I undertake, under my personal responsibility, to avoid during the entire trial period the occurrence of pregnancy, because it cannot be excluded the possibility of malformations of the unborn.

ETHICAL COMMITTEE DECISION: SUBSTANTIAL AMENDMENT APPROVED

It acknowledges that the Ethics Committee has examined the following substantive amendment to the Study Protocol:

- #1 Study Protocol including the amendment of 12 February 2014
- #1 Copy of Information Sheet for the patient dated 12 February 2014;
- #1 Copy of the Consent Form on February 12, 2014.

Il Presidente del Comitato Etico
Prof.ssa Graziella Filippini
