# Supplementary material for: Efficacy and Safety of Extracranial Vein Angioplasty in Multiple Sclerosis: A Randomized Clinical Trial
Source: JAMA Neurol. 2017 Nov 18;75(1):35–43. doi: 10.1001/jamaneurol.2017.3825 (PMC5833494; doi:10.1001/jamaneurol.2017.3825)
Supplement: Supplement 2. — eMethods. Information on the novel functional composite. eFigure. Study diagram for patients with secondary progressive multiple sclerosis. eTable 1. Magnetic resonance imaging findings at 0 to 6 months. eTable 2. Clinical and magnetic resonance imaging end points (changes at 12 months compared with baseline) according to treatment group in the 15 patients with secondary progressive multiple sclerosis (intention-to-treat population). [file jamaneurol-75-35-s002.pdf]

Supplementary Online Content

Zamboni P, Tesio L, Galimberti S, et al; the Brave Dreams Research Group. Efficacy and safety of extracranial vein angioplasty in multiple sclerosis: a randomized clinical trial. *JAMA Neurol*. Published online November 18, 2017. doi:10.1001/jamaneurol.2017.3825

eMethods. Information on the novel functional composite
eFigure. Study diagram for patients with secondary progressive multiple sclerosis
eTable 1. Magnetic resonance imaging findings at 0 to 6 months
eTable 2. Clinical and magnetic resonance imaging end points (changes at 12 months compared with baseline) according to treatment group in the 15 patients with secondary progressive multiple sclerosis (intention-to-treat population)
eReferences.

This supplementary material has been provided by the authors to give readers additional information about their work.

© 2017 Zamboni et al. *JAMA Neurology.*

eMethods. Information on the novel functional composite

The walk ratio was estimated as a summary index of neural control of gait. The walk ratio is the ratio of stride length (in mm) to step frequency (per minute). This index largely independent of speed. Higher values indicate better neural control of gait. The ratio is usually standardized to the person's height[1], however no significant differences have been ever found between crude and standardized estimates, so that crude values only were used in Brave Dreams.

The maximum excursion endpoint was used as measure of standing balance. The maximum excursion is one of the tests performed with the Balance Master instrument.[2] While standing, the person looks at a computer screen about 1.8 m away. The screen shows a human silhouette and indicates different directions of leaning by lighting up one of eight squares surrounding the silhouette (each 45 degrees from the other). On an acoustic 'go' signal, the individual has to lean (as quickly as possible) in the direction indicated by the lit up square. He/she does not usually take a straight line path toward the target. At 8-second intervals another square (target) is lit up in turn and the person has to lean in that direction. An on-screen trace displays the horizontal path of the body's centre of mass during the leaning. The amount of leaning achieved is given as a percentage of the straight-line distance from the person to the target. The result is the mean leaning toward each of the eight target. The nearer the result is to a 100, the better the balance.

The box and block test[3] was selected to measure manual dexterity. This test assesses visual and sensory motor control of the whole upper limb, but emphasises wrist and finger mobility. The person is asked to move 2.5-cm-cubed blocks, as fast as possible, from one compartment of a box to another compartment of equal size. The two compartments are separated a 15.2 cm-high partition. One arm at a time is tested. The outcome is the number of blocks displaced in a minute: the higher the better. A change (over time) in the performance of test is a composite outcome. A person is considered improved if the improvement occurs on one side, the other side being unchanged; the outcome is worsened if worsening occurs on one side, other side being unchanged or worsened; the outcome is unchanged if no changes occur (on any side), and mixed if worsening occurs on one side and improvement on the other. A mixed outcome was never observed in the Brave Dreams trial.

The postvoid residual urine volume in the bladder was used as measure of control of micturition. This is the volume of urine remaining in the bladder after a voluntary micturition. It is measured with a small dedicated ultrasound scanner, and has precision of about 20 ml.[4] The lower the residual volume the better.

© 2017 Zamboni et al. *JAMA Neurology.*

Visual acuity was tested on standard ETDRS optotype charts with 100%, 2.5% or 1.25% contrast.[5] Each chart has 14 rows, with five capital letters per row. Letter size decreases from top to bottom. Lines are read from top to bottom. A line is considered read when at least three letters are correctly recognized. The outcome can be either the number of lines or the number of letters read at each contrast level: the more the better. In Brave Dreams we assessed number of lines. A patient is considered improved or worsened if a significant change occurred in at least one contrast level, with no change at other levels. The patient is unchanged if no change occurs any contrast level. It is possible improve at one contrast level and worsen at another to generate a mixed outcome. This did not happen in Brave Dreams.

For all tests, normative cut-offs were obtained from the literature. A key issue was defining significant and clinically meaningful changes. For the walking, balance and manual dexterity measure a formal "minimal real difference" was available.[6] For postvoid residual urine volume, changes greater than 30 ml were considered clinically significant for residual volumes to 200 ml; changes greater than 50 ml clinically significant for residuals volumes above 200 ml. For the ETDRS visual acuity charts, a previous MS trial[7] considered that a change at least two lines was clinically significant; this threshold was adopted in Brave Dreams.

© 2017 Zamboni et al. *JAMA Neurology.*

eFigure. Study diagram for patients with secondary progressive multiple sclerosis

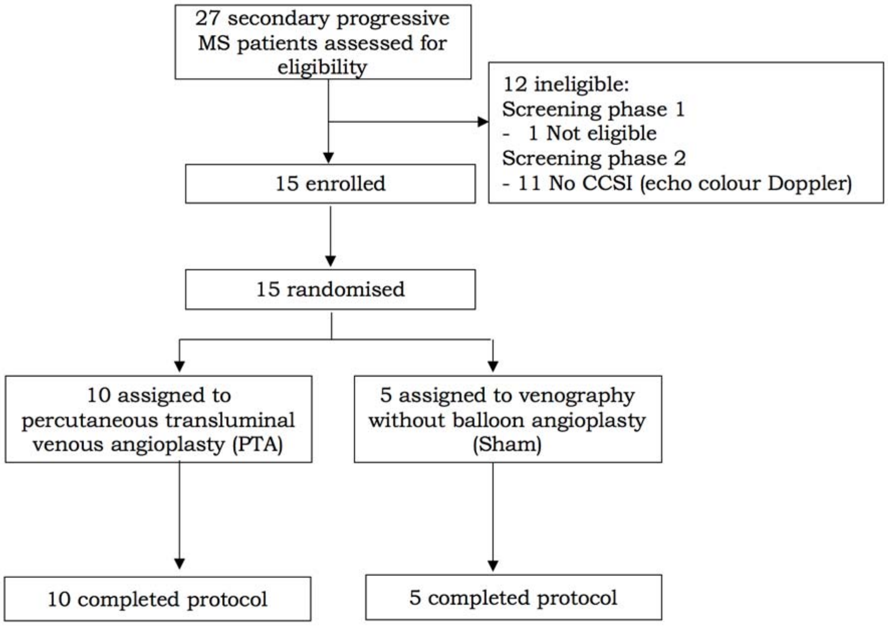

© 2017 Zamboni et al. *JAMA Neurology.*

eTable 1. Magnetic resonance imaging findings at 0 to 6 months

Measure	Months 0-6			
	**PTA (n = 73)**	**Sham (n = 37)**	***P* Value**	**Adjusted *P* Value[a]**
New combined brain lesions[b]				
No. of lesions, mean (SD)	0.97 (3.58)	0.76 (1.37)	NA	NA
Mean lesion ratio (95% CI)[c]	1.28 (0.46-3.57)		.63	>.99
Median (range)	0 (0-27)	0 (0-5)	NA	NA
Patients free of lesions, No. (%)	50 (71.4)	22 (66.7)	NA	NA
Lesion free, OR (95% CI)[d]	1.48 (0.65-3.37)		.47	>.99
New or enlarged T2 lesions				
No. of lesions, mean (SD)	0.77 (2.58)	0.55 (1.12)	NA	NA
Mean lesion ratio (95% CI)[c]	1.40 (0.48-4.16)		.53	>.99
Median (range)	0 (0-17)	0 (0-5)	NA	NA
Patients free of lesions, No. (%)	52 (74.3)	24 (72.7)	NA	NA
Lesion free, OR (95% CI)[d]	1.34 (0.58-3.12)		.64	>.99
Gadolinium-enhancing T1 lesions[e]				
No. of lesions, mean (SD)	0.69 (3.41)	0.39 (0.97)	NA	NA
Mean lesion ratio (95% CI)[c]	1.74 (0.37-8.09)		.48	.96
Median (range)	0 (0-27)	0 (0-5)	NA	NA
Patients free of lesions, No. (%)	61 (87.1)	25 (75.8)	NA	NA
Lesion free, OR (95% CI)[d]	2.44 (0.82-7.22)		.15	.30

Abbreviations: OR, odds ratio; PTA, percutaneous transluminal angioplasty.

[a]*P* values adjusted for multiplicity using Hommel method.

[b]New combined lesions included new lesions on T2-weighted images, preexisting lesions enlarged by >30% on T2-weighted images, and gadolinium-enhancing lesions in T1-weighted images of preexisting lesions (no enlarged T2 lesions were observed).

[c]Estimated effect of PTA on number of lesions; negative-binomial model was used to derive *P* values.

[d]Estimated effect of PTA on proportion lesion-free patients; χ^2 test was used to derive *P* values.

[e]Sixty-nine patients in the PTA group and 33 in the sham group had data on gadolinium-enhancing lesions.

© 2017 Zamboni et al. *JAMA Neurology.*

eTable 2. Clinical and magnetic resonance imaging end points (changes at 12 months compared with baseline) according to treatment group in the 15 patients with secondary progressive multiple sclerosis (intention-to-treat population)

	**PTA (n=10)**	**Sham (n=5)**
Composite functional endpoint		
Improved	4 (40.0%)	3 (60.0%)
Stable	3 (30.0%)	1 (20.0%)
Worsened	1 (10.0%)	0 (0.0%)
Mixed	2 (20.0%)	2 (20.0%)
MRI endpoint		
New lesions[a]		
N lesions, mean (SD)	0 (0)	0.80 (0.84)
Median (min-max)	0 (0 to 0)	1 (0 to 2)
N patients free of these lesions (%)	10 (100.0%)	2 (40.0%)
New or enlarged T2-weighed lesions		
N lesions, mean (SD)	0 (0)	0.60 (0.89)
Median (min-max)	0 (0 to 0)	1 (0 to 2)
N patients free of these lesions (%)	10 (100.0%)	3 (60.0%)
Gadolinium-enhancing lesions		
N lesions, mean (SD)	0 (0)	0.40 (0.55)
Median (min-max)	0 (0 to 0)	1 (0 to 1)
N patients free of these lesions (%)	10 (100.0%)	3 (60.0%)

[a]Combined lesions comprise new or enlarged lesions in T2 plus gadolinium-enhancing lesions in T1.

© 2017 Zamboni et al. *JAMA Neurology.*

eReferences.

1. Rota V, Perucca L, Simone A, Tesio L. Walk ratio (step length/cadence) as a summary index of neuromotor control of gait: application to multiple sclerosis. Int J Rehabil Res 2011;34:265-9.

2. Tesio L, Rota V, Longo S, Grzeda MT. Measuring standing balance in adults: reliability and minimal real difference of 14 instrumental measures. Int J Rehabil Res. 2013;36,4:362-374

3. Goodkin DE, Priore RL, Wende KE, Campion M, Bourdette DN, Herndon RM, et al. Comparing the ability of various compositive outcomes to discriminate treatment effects in MS clinical trials. The Multiple Sclerosis Collaborative Research Group (MSCRG). Mult Scler. 1998;4,6:480-486

4. Kalsi V, Fowler CJ. Therapy insight: bladder dysfunction associated with multiple sclerosis. Nat Clin Pract Urol. 2005;2,10:492-501.

5. Mowry EM, Logiudice MJ, Daniels AB, Jacobs DA, Markowitz CE, Galetta SL, Nano-Schiavi ML, Cutter GR, Maguire MG, Balcer LJ. J Neurol Neurosurg Psychiatry. 2009; 80,7:767-772

6. Tesio L. Outcome measurement in behavioural sciences: a view on how to shift attention from means to individuals and why. Int J Rehabil Res, 2012; 35,1:1-12

7. Balcer LJ, Frohman EM. Evaluating loss of visual function in multiple sclerosis as measured by low-contrast letter acuity. Neurology. 2010;27;74 Suppl 3:S16-23

© 2017 Zamboni et al. *JAMA Neurology.*
